# A Protocol for the Secure Linking of Registries for HPV Surveillance

**DOI:** 10.1371/journal.pone.0039915

**Published:** 2012-07-02

**Authors:** Khaled El Emam, Saeed Samet, Jun Hu, Liam Peyton, Craig Earle, Gayatri C. Jayaraman, Tom Wong, Murat Kantarcioglu, Fida Dankar, Aleksander Essex

**Affiliations:** 1 Electronic Health Information Laboratory, Children’s Hospital of Eastern Ontario Research Institute, Ottawa, Ontario, Canada; 2 Paediatrics, University of Ottawa, Ottawa, Ontario, Canada; 3 School of Electrical Engineering and Computer Science, University of Ottawa, Ottawa, Ontario, Canada; 4 Institute for Clinical Evaluative Sciences, Toronto, Ontario, Canada; 5 Public Health Agency of Canada, Ottawa, Ontario, Canada; 6 Computer Science, University of Texas at Dallas, Dallas, Texas, United States of America; 7 Department of Epidemiology and Community Medicine, University of Ottawa, Ottawa, Ontario, Canada; 8 Division of Infectious Diseases, University of Ottawa, Ottawa, Ontario, Canada; The Centre for Research and Technology, Hellas, Greece

## Abstract

**Introduction:**

In order to monitor the effectiveness of HPV vaccination in Canada the linkage of multiple data registries may be required. These registries may not always be managed by the same organization and, furthermore, privacy legislation or practices may restrict any data linkages of records that can actually be done among registries. The objective of this study was to develop a secure protocol for linking data from different registries and to allow on-going monitoring of HPV vaccine effectiveness.

**Methods:**

A secure linking protocol, using commutative hash functions and secure multi-party computation techniques was developed. This protocol allows for the exact matching of records among registries and the computation of statistics on the linked data while meeting five practical requirements to ensure patient confidentiality and privacy. The statistics considered were: odds ratio and its confidence interval, chi-square test, and relative risk and its confidence interval. Additional statistics on contingency tables, such as other measures of association, can be added using the same principles presented. The computation time performance of this protocol was evaluated.

**Results:**

The protocol has acceptable computation time and scales linearly with the size of the data set and the size of the contingency table. The worse case computation time for up to 100,000 patients returned by each query and a 16 cell contingency table is less than 4 hours for basic statistics, and the best case is under 3 hours.

**Discussion:**

A computationally practical protocol for the secure linking of data from multiple registries has been demonstrated in the context of HPV vaccine initiative impact assessment. The basic protocol can be generalized to the surveillance of other conditions, diseases, or vaccination programs.

## Introduction

The human papillomavirus (HPV) is one of the most prevalent sexually transmitted viral infections in the world [1]. Persistent infection with oncogenic high-risk HPV types, in particular 16 and/or 18, accounts for the majority of cervical cancer and is associated with oral, vulvar, vaginal, penile and anal intraepithelial neoplasia and cancer [2]. HPV is also the cause of external genital warts, with over 90% attributable to low-risk HPV types 6 and 11 [2]. HIV co-infected individuals are at greater risk of developing rarer and/or more aggressive forms of cancer such as anal and penile carcinoma as well as genital warts [2].

Since 2007 an effective preventive quadrivalent vaccine has been available in Canada that protects against low risk (non-oncogenic) types 6 and 11, and high risk (oncogenic) types 16 and 18; in 2010 a bivalent second vaccine against types 16 and 18 was approved for use. Currently, publicly funded school-based HPV immunization programs have been implemented for girls in all 13 Canadian jurisdictions. However, program details, such as school grade(s) in which the vaccine is offered and whether or not there is a catch-up program, vary by province/territory. While the vaccine has the potential to substantially reduce costs associated with screening and treatment and to reduce the overall HPV-related disease morbidity/mortality burden, the long-term and population-level effectiveness of this vaccine are not known. HPV surveillance and research are necessary in order to understand the vaccine’s impact on population health and to inform policy decisions concerning the allocation of health care resources. Detailed baseline information on the distribution and determinants of HPV types, variation by geographic location and risk behaviour is not available for all regions in Canada. Noting that vaccine impact on cancer incidence will not be measurable in a vaccinated population for another generation, we need to monitor HPV type distribution, changes in sexual behaviour, and associations with cytological abnormalities (including pre-cancerous lesions), as well as anogenital warts in the short term.

One of the proposed mechanisms to address the short- and long-term objectives to assess the impact of the HPV vaccine introduction is via the linkage of population-based databases (registries) on cancer, cervical screening, health care services, and immunization. Certain jurisdictions, for example, Manitoba, have robust population-based registries; others are in the early stages of developing such systems. Regardless of the maturity of such registry-based systems, data linkages between registries can only be conducted in an environment that is responsive to patient privacy concerns.

Statutes in Canadian jurisdictions permit the reporting of personal health information (PHI) [3] for public health purposes without patient consent. Similarly, the US Health Insurance Portability and Accountability Act (HIPAA) Privacy Rule permits the disclosure of PHI to a public health authority without patient authorization [4]–[10]. However, in general, the public is more comfortable with their health information being used for secondary purposes if it is de-identified at the earliest opportunity [11]–[18] and in practice providers and data custodians have been reluctant to disclose identifiable patient information to public health even when permitted by legislation [19]–[22]. Such reluctance can be overcome if patient consent to disclose the data for public health purposes is sought. However, there is compelling evidence that requiring explicit consent can bias data sets because consenters and non-consenters differ on important demographic and socio-economic characteristics [23]–[25].

In this paper we present a protocol for the secure linking and surveillance of patient records in different registries where the sharing of identifying patient information is not possible, either because the registries are not set up to allow for such linkages or because the custodians of the registries are not authorized to link data between them due to patient privacy concerns. The proposed protocol will allow a public health unit (PHU) to compute relevant statistics from linked data on an on-going basis while providing strong patient privacy guarantees.

## Methods

### Motivating Example

While linking data registries when identifiable information about the patients cannot be shared is a general problem, we consider it within the context of an HPV surveillance example to motivate and illustrate our solution. We assume that there are two registries. One registry contains demographic information about the population, and the second registry contains the results of HPV-associated tests. For example, the former can be a large practice, a hospital, or a vital statistics registry. The latter can be at private or public laboratories at the local or provincial level.

As an example, should the PHU wish to investigate the relationship between HPV test results and ethnicity, the relationship can be expressed as a chi-square test, an odds ratio test, or a relative risk. If HPV test results are captured in one registry and ethnicity in another (as is typically the case), any analyses require that the records in the two registries be linked. Table 1 shows the contingency table that we need to construct to investigate the association between ethnicity and the results of an HPV test. The cells of the table are the counts of patients. We refer to the two registries as R1 (with HPV screening data) and R2 (with ethnicity data).This example can be extended to multiple dimensions without loss of generality, but we will use this simple 2×2 example to discuss previous work in this area and to illustrate our secure linking protocol.

**Table 1:** Example of a contingency table for which we want to compute a bivariate relationship.

		Any HPV (R1)	
		−ve	+ve
**Ethnicity (R2)**	Aboriginal	n_11_	n_12_
	White	n_21_	n_22_

The two registries will not contain exactly the same patients, but there is expected to be an overlap between them. This means that not all patients in R1 will have records in R2 and vice versa.

The two registries hold one or more common linking fields on all of their patients. We assume that these linking fields are direct identifiers, such as a health insurance card number and/or a social insurance number. In practice, multiple fields can be concatenated or encoded to create a single identifier used for linking. Also, note that these direct identifiers do not need to be numeric but can be strings, dates or categorical values. For example, in a Canadian context the date of birth, postal code, and gender uniquely identify approximately 99% of adults living in urban area [26], making that combination of commonly collected demographic information suitable for linking purposes.

For simplicity, and without loss of generality, we will refer to a single linking field in Table 1. By comparing the two registries on the linking field it would be possible to match their records with certainty. Because the linking field is a direct identifier, it can be used to determine the identity of the patients, and would therefore be considered personally identifying information.

### Requirements for a Secure Linking Protocol

Based on the practical realities of privacy problems experienced by an actual PHU, we have formulated five requirements for a protocol to link the data in the two registries. We will examine each of these requirements in turn to illustrate the strengths and weaknesses of the various approaches that have been proposed:

- **A1. The PHU cannot collect personal health information from the registries.** The registries cannot disclose identifiable health information to the PHU because of legislative constraints or because they have reservations about privacy.
- **A2. The protocol must not use a trusted third party (TTP).** A TTP would be an entity independent of the registries and the PHU, but would be able to access identifiable information about the patients. While it is possible to use a TTP to link the data from the two registries, there are pragmatic challenges to consider. First, the custodians of both registries need to be able to share the data with the TTP, and this may be challenging if the registries are within different organizations or jurisdictions, and each wants the TTP to be ‘housed’ within their organization or jurisdiction. For example, if the source registries are in different jurisdictions but cover the same patient population (e.g., provincial and federal) they may not agree on who the TTP should be. Second, the registries may not have the authority to disclose personal information to a third party without patient consent, even if it is for the purpose of linking information and the data remain within the jurisdiction.
- **A3. The two registries must not have to trust each other.** It must not be necessary for the registries linking their data to trust each other. This lack of trust may be driven by security or legal concerns. For example, trust is necessary if the two registries need to share a secret key, in which case each registry must trust the other one will protect the key since a key compromise would endanger the information held by both registries. Furthermore, the registries may not have the authority to share identifiable patient data amongst themselves without patient consent. For example, the two registries may be within two different government departments and there is no legal basis for sharing data between them.
- **A4. No new information can be discovered about patients in any one registry due to the linking exercise.** Any matching protocol must not allow a registry to discover new information about its patients that is gained from the linking with the other registry. It is often the case that a registry is not able to collect new information without consent or additional authorization. This is a common requirement in the privacy-preserving computation literature where parties collaborating in a computation must not learn something new due to their participation [27], [28].
- **A5. A security compromise at the site of any party involved in the protocol must not reveal the identity and any sensitive information of any patients.** In addition to the registries that are the data sources for linking, other parties involved in the secure linking protocol should not hold any PHI. This will ensure that if the security of these other parties is compromised that no PHI will be disclosed.

The above requirements have been implicitly acknowledged in the literature in that different protocols have been developed to address subsets of them. In the following review we examine how well these requirements have been met.

### Data Linking Architectures and Protocols

**Figure 1:**
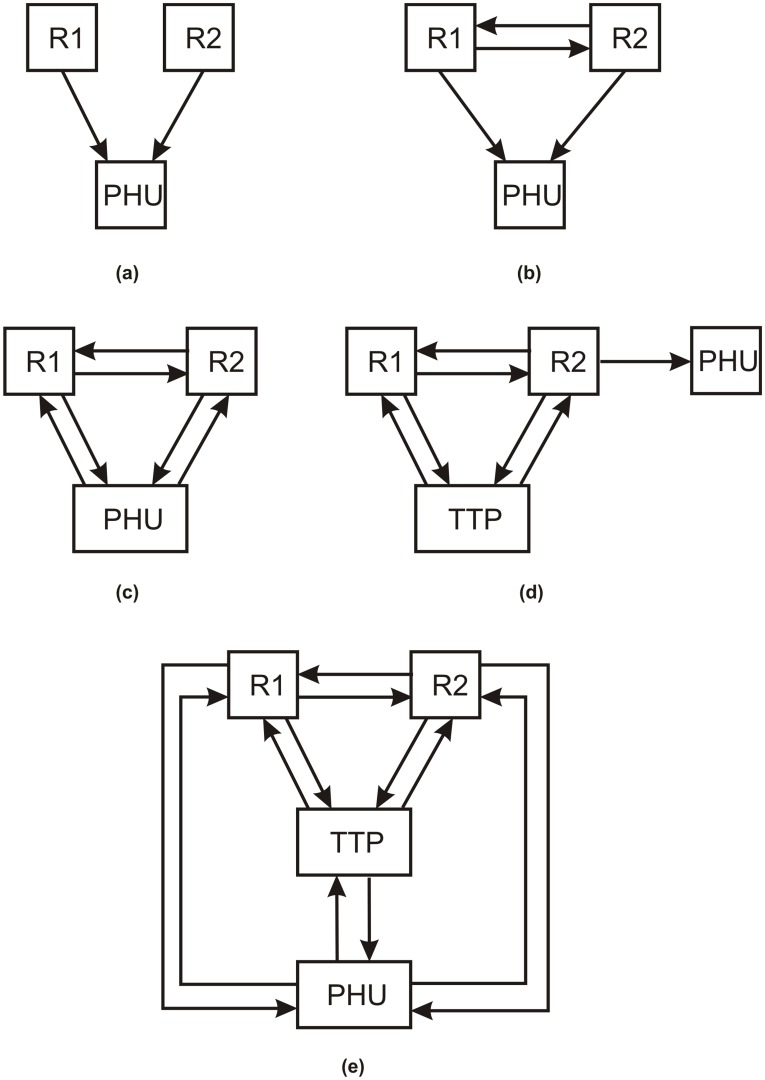
Different architectures in the literature for linking two registries.

Current data linking protocols can be classified into one of the five architectures shown in Figure 1. The simplest architecture is (a), where both registries provide their raw data to the PHU, which then performs the linking. This means that the registries provide the PHU with the linking fields, ethnicity, and the HPV results. This protocol does not meet requirements A1 and A5. For A1, the PHU would get identifiable patient information, and for A5 a security compromise at the PHU would reveal the identity and sensitive information of the patients.

Under architecture (b) in Figure 1, R1 can give the linking fields for all of its patients to R2 to link with its own data. R2 links the data and generates new unique keys for all of the records, which it sends back to R1. Then both registries send their ethnicity and HPV data with the unique keys separately to the PHU, which can re-link the data and create the contingency table in Table 1. However, with this protocol, a registry may discover new information about its patients. For example, R2 may discover which one of its patients has been tested for HPV. This would fail on requirement A4. While the fact of being tested for HPV may not seem like a major breach if testing is common, consider situations where a registry only holds information about those receiving treatment for drug addiction and substance abuse, or individuals who receive social assistance. In such a case knowing that an individual exists in a particular registry could reveal highly sensitive information. Furthermore, this approach does not meet requirement A3. Finally, the PHU could potentially re-identify individuals because when the data is cross-tabulated as in Table 1 there may be small cells in the contingency table. Small cells can allow an adversary to re-identify patients. We examine this situation in more detail below.

Consider Table 2, which covers a known population. The Aboriginal individual who was not HPV positive will know that all of the other Aboriginal individuals in the data set tested positive. In this case an individual in the data can reveal information about all other individuals in the data. Table 3 would allow an external user to know that all Aboriginal individuals in the data set tested positive.

**Table 2:** Example of a contingency table for which there is a high identity disclosure risk within the population.

		Any HPV	
		−ve	+ve
**Ethnicity**	Aboriginal	1	11
	White	50	15

If the data in Table 2 and Table 3 does not represent the whole or a known population, there is still a risk of re-identifying individuals. This would occur, for example, if the data set is a sample. For Table 2, if one can estimate that there was only a single individual in the cell ‘Aboriginal and Negative’ in the whole population (i.e., can estimate that the sample ‘unique’ is also a population ‘unique’), then the same re-identification risk as for small cells exists. Similarly, for Table 3, if one can determine that there were indeed only five positive Aboriginals in the population then the same re-identification risk exists as for small cells. It is possible to estimate the cell size for the population using sample data [29].

**Table 3:** Example of a contingency table for which there is a high identity disclosure risk from an external attacker.

		Any HPV	
		−ve	+ve
**Ethnicity**	Aboriginal	0	5
	White	50	15

One possible solution is to develop a protocol to suppress the small cells in the contingency table before it is disclosed to the PHU. However, it would not be possible to compute associations on tables with suppressed cells. In addition, by executing additional queries on the data it would be possible to reconstruct the suppressed cells. For example, consider Table 4 which had a small cell suppressed. Now the PHU can execute another query shown in Table 5 that does not have small cells on the same data (assuming a minimal cell size of 5). Because the marginal totals for the ethnicity in the two tables are expected to be the same, it would be possible determine that the value of the suppressed cell in Table 4 was 1. As another example, consider Table 6 which had a small cell suppressed. Another query, shown in Table 7, with gender instead of ethnicity does not have small cells and reveals the marginal totals for the HPV results, which then reveals that the suppressed cell in Table 6 is of size zero. More generally, iterative algorithms can be used to determine the exact value or a narrow value range for suppressed cells if the marginal totals are known [30].

**Table 4:** Example of contingency table with suppressed cells.

		Any HPV	
		−ve	+ve
**Ethnicity**	Aboriginal	–	11
	White	50	15

**Table 5:** Example of contingency table that would reveal the contents of the suppressed cells in Table 4.

		Age	
		<20	>20
**Ethnicity**	Aboriginal	6	6
	White	50	15

**Table 6:** Example of contingency table with suppressed cells.

		Any HPV	
		−ve	+ve
**Ethnicity**	Aboriginal	–	5
	White	50	15

**Table 7:** Example of contingency table that would reveal the contents of the suppressed cells in Table 6.

		Any HPV	
		−ve	+ve
**Gender**	Male	25	2
	Female	25	18

Therefore, as long as multiple tables can be generated it cannot be guaranteed that cell suppression would work. This is a known inference problem in statistical databases [31].

Secure protocols following architecture (b) have been proposed [27], [32]–[38]. These require collaboration among the registries and at the end of their joint computations the contingency table would be shared with the PHU by one or both of the registries. However, they would all be prone to the small cells problem noted above, and the two registries would still need to trust each other since in many of these protocols one of the registries would end up with the contingency table (requirement A3). In addition, some protocols will only work with three or more registries [34], [38], and would therefore not be applicable in the simplest case of two registries.

A slight modification is architecture (c) where the PHU actively participates in the matching and the computations needed rather than being just a recipient of data. One approach is for the two registries to agree on a secret random value and concatenate it to the linking variables, and then hash this concatenated value. The hashed values are then sent to the PHU from each registry [39], [40]. The PHU would match the hashed values from the two registries, count the number of matching hashes, and compute the cell values in the contingency table of Table 1. A number of other cryptographic protocols have been developed that are suitable for this architecture [41]–[45]. These protocols also do not meet the same requirements as the ones following architecture (b) in that they can reveal identifying information to the PHU through small cells and require both registries to trust each other.

Some protocols use a TTP to participate in the linking instead of providing the data to the PHU as illustrated in architecture (d). The TTP would not obtain the contingency table. Instead, the contingency table would be computed from one or both registries and this information would be transmitted to the PHU. One protocol requires the registries to send hashed values to the TTP who then performs the linking [46]. However, this is prone to a dictionary attack, which is when an adversary tries all possible input values until one hashes to the same value. More secure protocols have been proposed [47], [48], but these have the same disadvantages as those following architecture (c), as well as requiring a TTP and being vulnerable to collusion between the TTP and one of the registries. Furthermore, if a TTP’s security is compromised then this would result in a significant breach affecting both registries.

The final architecture illustrated in panel (e) also requires a TTP. In one deployment of this architecture in Wales, the demographic information is separated from the clinical information, and all of the data sources send the demographic information to a TTP who then performs probabilistic matching of the records on these variables, generates a new unique identifier for each record to allow linking, and sends the unique identifier back to the data sources [49], [50]. The data sources then provide the unique identifier with the clinical information to a databank accessed by external parties, such as researchers. In our case the databank would be housed in the PHU. Another protocol that does not use cryptographic techniques utilizes a TTP to run remote sub-queries at the registries and combine and return results back to the PHU [51]. One protocol hashes the linking variables, but also requires that the registries share a secret key [52]. A more secure protocol that utilizes bloom filters has been proposed, which also requires a TTP [53]. A stand alone probabilistic linking technique has been proposed which can be used within this architecture [54]. All of these protocols would not meet requirement A1 because they are vulnerable to the small cells problem that could leak personal information.

Even though various subsets of the requirements have been met in previous research, there have been no protocols developed that address all of the requirements, which is the main contribution of this paper.

### Principles and Techniques

As background, we present a set of design elements and building blocks that we integrated into our final protocol.

### Using A Semi-Trusted Third Party

In our proposed protocol we use a semi-trusted third party (sTTP). This is a commonly used term to describe a party who would not be able to obtain personal information about the patients, even if it tried to do so. Therefore, there is no risk that patient privacy would be breached, making it unnecessary to fully trust that third party. The only requirement on the sTTP is that they execute the linking protocol faithfully. The utilization of an sTTP in a secure linking protocol would allow us to meet all of the requirements posited earlier. Utilization of an sTTP is a weaker trust requirement than requiring a trusted-third party, who would be able to obtain personal information if it wanted to (hence it needs to be fully trusted). Furthermore, a data breach from an sTTP would not compromise any PHI.

To ensure that the third-party need only be semi-trusted, we propose to compute the statistics that are needed by the PHU directly rather than disclose a contingency table to the PHU. The statistics we will use are the omnibus chi-square test, the odds ratio and its confidence interval, and the relative risk and its confidence interval. The remainder of the paper describes such a protocol while meeting all of the requirements.

### Commutative Hash Function

A hash function transforms an input value 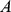 to an output value 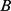 such that the 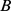 value is unique to 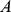 and it is not possible to obtain 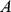 from 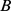. The input 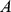 would typically consist of a message to be hashed and a key. A commutative hash function 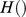 has the additional property that:
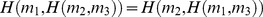
(1)This means that multiple applications of the hash function in different order will produce the same results. A detailed discussion of a commutative hash function based on the discrete log that is suitable for the problem of matching records from multiple registries is presented in Appendix S1.

### Secure Computation

Privacy-preserving computation protocols often utilize Secure Multi-party Computation (SMC) [55], [56]. SMC computes the final result in a secure way among multiple parties. Cryptographic and other tools are often used among two or more parties to jointly and securely compute one or more functions using their own private inputs. By using this approach, the final result is the same as that in the corresponding non-secure algorithm, and thus the main trade-off is between security and efficiency. SMC methods have been used previously to define secure disease surveillance protocols [57].

One of the popular encryption techniques used in privacy-preserving methods is homomorphic encryption. In this type of cryptosystem, one operation on the plaintexts will be mapped to another, or even the same, operation on the ciphertexts. For instance, in Paillier [58] encryption, for any two plaintext messages 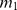 and 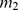 and their encryption 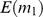 and 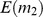, the following equation is satisfied:
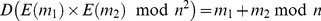
(2)where 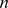 is a product of two large prime numbers, and 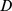 is the decryption function. Therefore, in this type of cryptosystem addition of the plaintexts is mapped to multiplication of the corresponding encrypted values. The Paillier cryptosystem also allows a limited form of the product of an encrypted value:
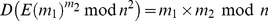
(3)which allows an encrypted value to be multiplied with a plaintext value to obtain their product.

Another property of Paillier encryption is that it is probabilistic. This means that it uses randomness in its encryption algorithm so that when encrypting the same message several times it will, in general, yield different ciphertexts. This property is important to ensure that an adversary holding a public key would not be able to compare an encrypted message to all possible encrypted counts from zero onwards and determine what the original plaintext value is.

In our protocol we use two sub-protocols based on the Paillier cryptosystem that to perform the intermediate calculations [59]: secure two-party addition and secure two-party multiplication.

Secure two-party addition allows any two parties to jointly add two integers together without either party revealing the value of the individual integer to the other, and without sharing the sum. Each party ends with a partial result (private output values). The two parties, 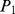 and 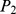, each has her own private integer value, 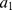 and 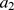 respectively. They obtain their own private output values, 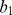 and 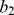, such that:
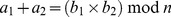
(4)Secure two-party multiplication allows the two parties to jointly multiply two integers without revealing the values of these integers or the resulting product to each other. For two parties, 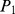 and 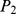, they compute their own private output values, 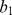 and 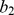, using their private input values, 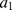 and 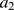 such that:



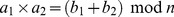
(5)


### The Secure Linking Protocol

There are three actors in our protocol as follows:

**Registry.** The custodians of the data that need to be linked. In our example we assume two registries but this can be easily extended without loss of generality.

**Aggregator.** The aggregator is a semi-trusted third party who can securely compute the statistics on the contingency table and sends the result back to the PHU. We assume there are two aggregators.

**PHU.** Defines which data elements are required and receives the final result of the analysis.

The protocol has three phases: (a) request, (b) matching, and (c) analysis.

### Request Phase

The protocol is initiated each time an analysis needs to be performed. For example, if the PHU wants to investigate the relationship between HPV test results and ethnicity, then the protocol is initiated. The completion of the protocol results in the production of the desired statistical result. In our example we assume that the PHU wishes to compute the odds ratio on the 2×2 contingency table. If the PHU then wants to investigate another relationship using the same two registries or different registries, say the relationship between HPV vaccination and HPV results, then the protocol would be initiated again.

**Figure 2:**
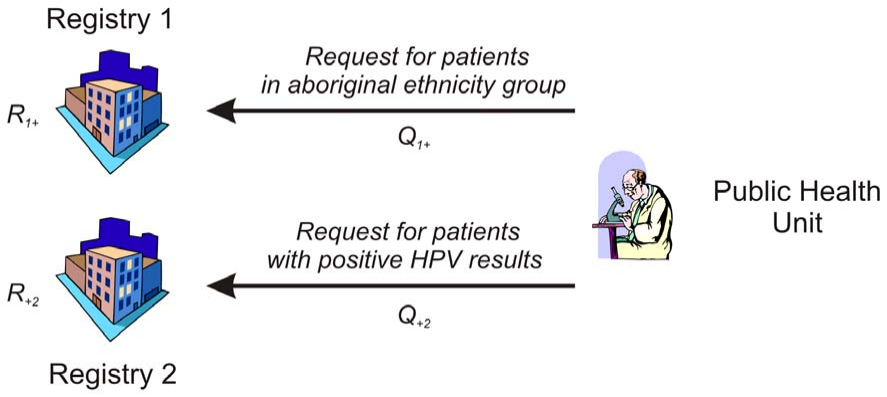
The public health unit sends a query for a particular cell within the desired contingency table.

The PHU then sends four different queries to the registries, one for each row and column in the contingency table. Each query has a unique identifier, 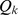, where the value of 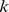 indicates the column or row in the contingency table. For example, the query 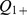 is for the Aboriginal patients. Another query, 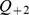 would be for patients with positive HPV results. This is illustrated in Figure 2. To compute statistics on a 2×2 table, four queries would need to be generated by the PHU with two targeted at each registry.

Upon receiving a query, each registry generates a random number only known to the registry, denoted by 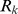. The random number is specific to each query.

### Matching Phase

Each registry would respond with a value for each patient matching the query. A registry would select an Aggregator at random to respond to. Let’s say that the direct identifier (linking field) for a patient from Registry 1 is denoted by 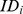 and the linking field for a patient from Registry 2 is denoted by 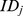. Registry 1 sends the hash value 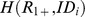 to the Aggregator.

**Figure 3:**
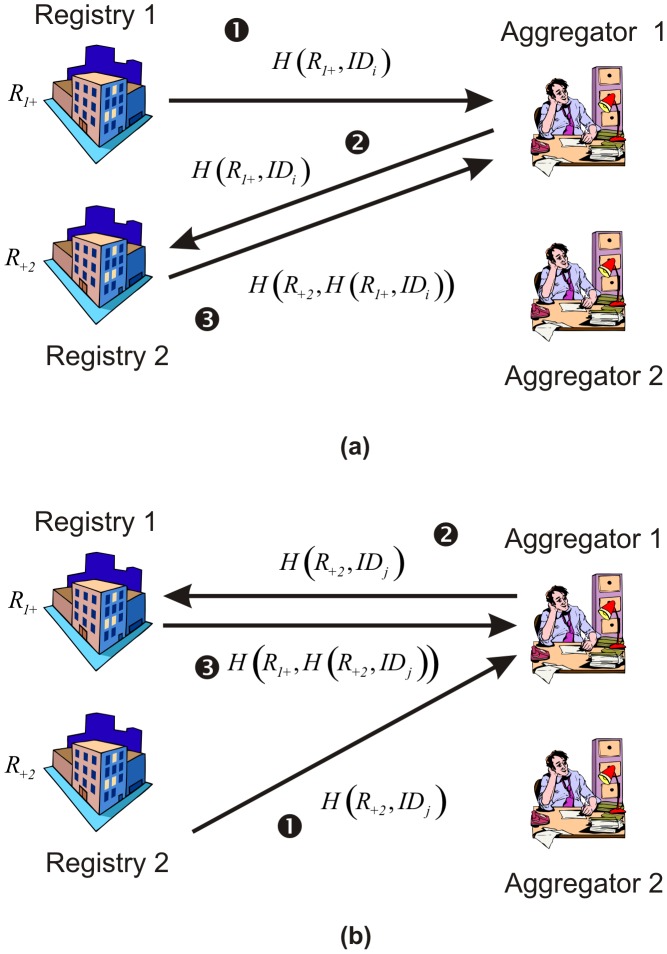
An example showing how a registry responds for a request for counts. A sequence of messages is generated for each patient.

In the example in Figure 3, Registry 1 sends this value to Aggregator 1. Aggregator 1 was chosen randomly by the registry and Registry 1 may send the value for the next patient to Aggregator 2. Because this is a hashed value and Aggregator 1 does not know the value of 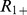, Aggregator 1 would not be able to determine the 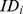 value. Aggregator 1 then forwards that information to Registry 2, which hashes that value and sends it back as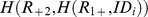. Aggregator 1 would then store that value.

Registry 2 would also respond with a message for every patient which satisfies the query. In the example of Figure 3, Registry 2 also selects Aggregator 1. By going through the same sequence of messages Aggregator 1 also gets 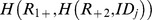 for the patient from Registry 2. If the same patient exists in both responses from Registry 1 and Registry 2, then 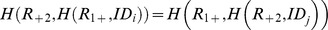 and Aggregator 1 would be able to determine that the same patient appeared in both registries within the cell making up the intersection of the queries. This is illustrated in Table 8. In this case Aggregator 1 matched two patients, and therefore its matched count would be 2.

**Table 8:** Example of the matching performed by Aggregator 1 and Aggregator2 based on the hash values that they receive.

Aggregator 1 Matching Table	
Registry 1	Registry 2
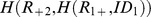	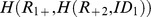
	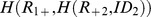
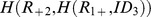	
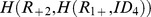	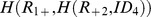
**Aggregator 2 Matching Table**	
**Registry 1**	**Registry 2**
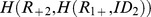	
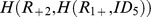	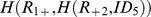
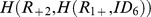	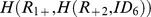
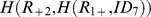	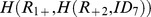

Because each Registry selects an Aggregator at random, no Aggregator will have a total count for a particular cell. This ensures that neither Aggregator will know with certainty if the cell has a small count. Therefore, Aggregator 1′s count of 2 is not a complete count of all Aboriginals with positive HPV results. There are two patients who were not matched by Aggregator 1 and it is not possible for Aggregator 1 to know whether these two patients exist in Aggregator 2′s table. Aggregator 2 had 3 patients matched. Therefore, in total we have 5 matched patients. However, the patient with an ID of 2 would not be matched because its values are split between the two Aggregators. The patient with ID number 3 would not be matched because there is no information on the ethnicity of that individual in Registry 2.

Once both registries have sent all of their hashed values to the Aggregators, the Aggregators need to reconcile their lists. Specifically, Registry 1 may have sent its value for a patient to Aggregator 1 and Registry 2 sent its value for the same patient to Aggregator 2. Therefore, it is not possible for either Aggregator to know that the patient exists in that cell. In our example, reconciliation would reveal that the patient with ID 2 is also matched.

The two Aggregators need to reconcile their lists and compute the final counts for the cell. Aggregator 1 knows that it already has a set of 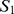 matching hashes and Aggregator 2 knows that it has a set of 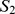 matching hashes. In our example we have 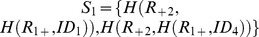 and 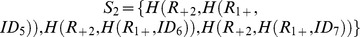. However, the union of these sets does not give us all of the matching patients.

Define a set of patients from Registry 1 who are not matched in either Aggregator:
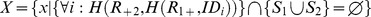
(6)and similarly from Registry 2:
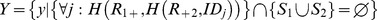
(7)Consider the notation for the contingency table in Table 9. Let us assume that we are computing the count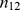. This consists of two counts, one from each of the aggregators, summed together 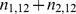. Below are the steps for computing 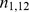 and 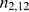:

**Table 9:** Notation for computing statistics.

		Any HPV	
		-ve	+ve
**Ethnicity**	**Aboriginal**	n_11_ = n_1,11_+n_2,11_	n_12_ = n_1,12_+n_2,12_
	**White**	n_21_ = n_1,21_+n_2,21_	n_22_ = n_1,22_+n_2,22_

1. On all 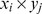 pairs, 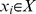 and 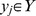, the Aggregators run a secure two-party subtraction. At the end of each secure two-party subtraction Aggregator 1 will have a value 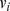 computed, and Aggregator 2 will have a value 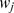 computed such that 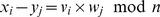 (see equation (4)). According to the secure two-party addition protocol, if Aggregator 1 initiates the protocol and 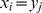, then 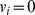. Otherwise if Aggregator 2 initiates the protocol and 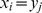, then 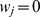. It does not matter which Aggregator initiates the protocol, but only one should do it to ensure that matches are not double counted. If either of 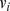 is zero or 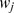 is zero, then the two values match.
2. Let 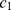 be the number of 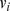
’s where 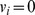, and let 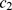 be the number of 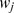
’s where 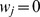.
3. Aggregator 1 computes:
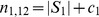
(8)and Aggregator 2 computes:
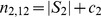
(9)Note that no single aggregator will know the value of 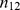 at the end of this protocol; it can only be computed by combining the values from both aggregators.

### Analysis Phase

**Figure 4:**
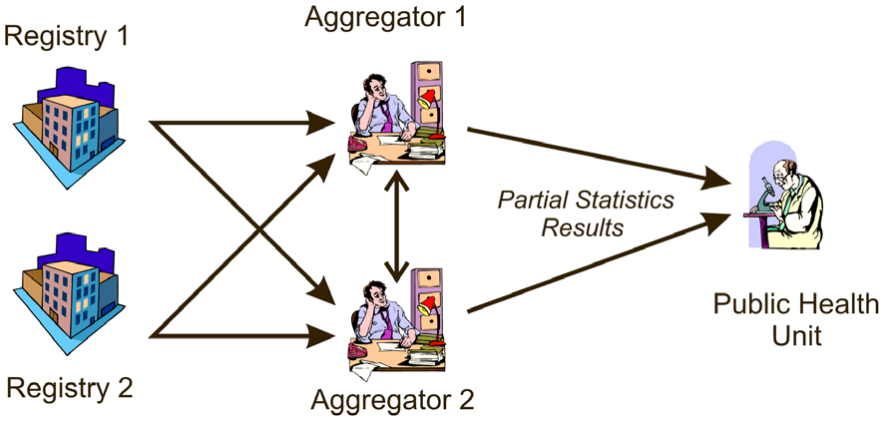
The flow of information between the Aggregators and the PHU.

The Aggregators can now jointly compute the appropriate statistics. As illustrated in Figure 4, the Aggregators then each send partial results of their statistical computations to the PHU, which combines the partial results to obtain the final result.

The count in each cell in the contingency table is split between the two Aggregators. Suppose we have a 2×2 contingency table as in Table 5. Below we go through the steps of calculation for the odds ratio. The computation of other bivariate statistics, such as chi-square, relative risk, and the confidence intervals for the odds ratio and relative risk, are described in Appendix S1.

In Table 9,
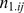
is available to Aggregator 1 and 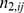 is available to Aggregator 2 for every 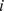 and 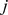. We can compute the odds ratio as follows:
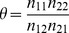
(10)which can be defined as:
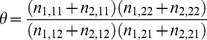
(11)To separate the above fraction such that each of the two Aggregators owns her final private values for the odds ratio we will apply secure two-party addition. In the equations below we will not show the “
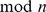
” to simplify the presentation. The steps of the protocol are as follows:

1. Aggregator 1 and Aggregator 2 run secure two-party additions for their following pairs:


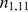
 and 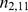 such that: 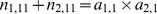



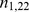
 and 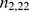 such that: 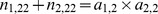



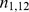
 and 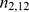 such that: 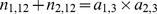



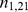
 and 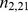 such that: 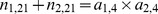


Therefore, the odds ratio will be converted to:



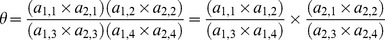



2. Aggregator 1 and Aggregator 2 then compute the two fractions 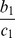 and 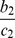, respectively, such that:



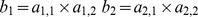


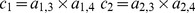



3. Aggregator 1 and Aggregator 2 send their private values 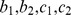 to the PHU. The PHU then computes 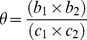.

A summary of the inputs and outputs, including for the statistics described in more detail in Appendix S1, are provided in Table10.

**Table 10:** Summary of the inputs and outputs for the building block and analysis protocols.

Protocol	Inputs	Outputs	Equation
Two-party addition	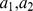	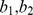	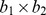
Two-party multiplication	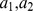	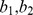	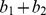
Odds Ratio	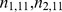	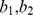	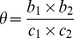
	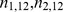	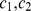	
	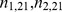		
	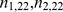		
Chi-square	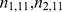	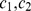	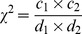
	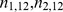	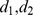	
	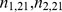		
	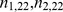		
Relative Risk	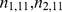	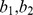	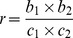
	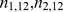	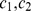	
	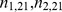		
	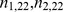		
Confidence Interval for Odds Ratio	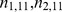	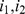	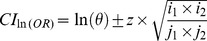
	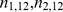	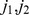	
	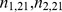		
	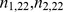		
Confidence Interval for Relative Risk	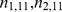	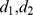	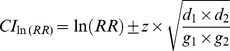
	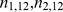	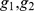	
	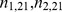		
	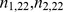		

### Empirical Performance Measurement

The challenge with secure computation protocols is that they are slower than non-secure ones. This makes the assessment of performance an important determinant of their practicability. We describe the communication costs in our analysis in Appendix S1. Here we focus on an empirical assessment of computation time.

The objectives of the evaluation were to determine: (a) the time to perform the computations under the different conditions, (b) how the protocol scales as the number of patients returned by the queries increases, and (c) how the protocol scales as the number of cells in the contingency table increases.

We assume that all queries return the same number 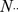 of patients. Let 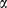 be the time it takes to perform a single encryption or decryption and let 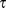 be the time it takes to perform a single commutative hash. Table 11 shows the total computation time range (matching and analysis tasks) for each of the statistics based on the detailed analysis in Appendix S1. Matching within the Aggregator is not considered as that computation time would be negligible compared to the computations requiring encryption and decryption. We consider the general case for the number of cells in the contingency table, which is denoted by 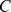 in Table 10. By examining Table 11, we would expect that computation time scales linearly with the number of patients and the number of cells.

**Table 11:** Summary of the computation time range for each of the statistics.

Statistic	Total Computation Time (max → min)
Chi-square	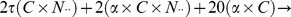 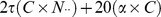
Odds Ratio	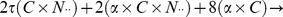 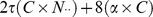
Relative Risk	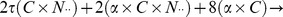 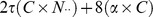

To evaluate the computation time performance of the protocol we empirically determined the values for 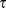 and 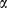 over 100 iterations for random values of the same integer size. The average hash time gave us an estimate for 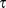. We then encrypted the hashed values and the average encryption time gave us 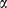. Using these two values we could determine the computation time for the implementation of the protocol. The timing was computed on a Windows machine running the XP operating system with an Intel dual core CPU running at 2.4 GHz and 2 GB of RAM. The key size used was 1024 bits.

We assume that 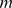 hashed values will be matched within an aggregator for all cells. In presenting the results, the value of 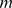 is expressed as a percentage of 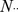 and varied from 0% to 100%, with the computation time in seconds calculated each time for a different number of patients. We let the total number of patients returned by each query requested from the two registries be 5,000, 10,000, 50,000, and 100,000. The value of 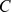 indicating the number of cells was varied from 4 to 16 in increments of 2.

## Results

The average time to perform an encryption using the Paillier cryptosystem is 1.721 ms, and decryption takes 1.882 ms. The secure multiplication and addition take 2.581 ms on average, and the average time to hash a value is 3.07 ms. The performance of the whole protocol was driven by the performance of the matching phase. The matching phase was driven by the size of the data set and the number of cells in the contingency table.

**Figure 5:**
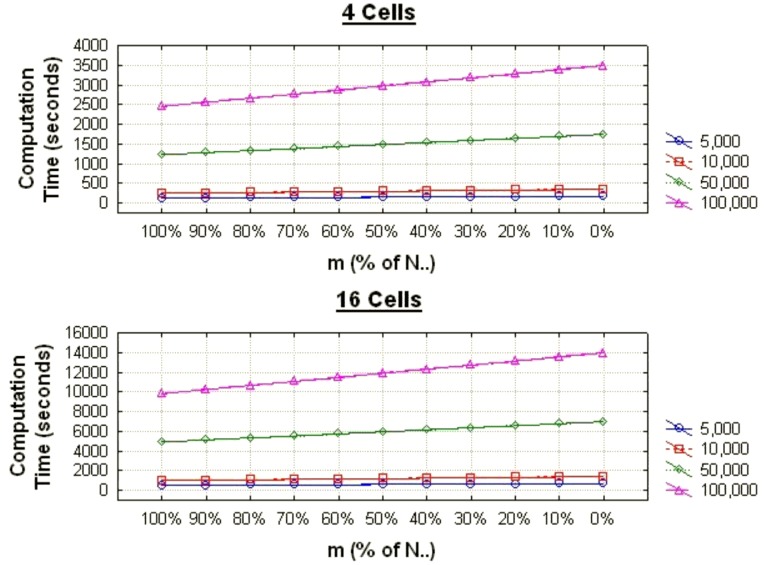
The average computation times for the chi-square test when the total number of records returned by the queries varies from the two registries are 5,000, 10,000, 50,000, and 100,000 for a 4 cell and a 16 cell contingency table.

Figure 5 illustrates the computation times for different percentage of matching records within each Aggregator, 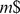. The computation time is smallest when 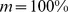 since that’s when all of the matching can be done within the Aggregator. For a contingency table with 4 cells and each query returning 100,000 patients, the total computation is just under one hour. With 16 cells it is just under 4 hours.

**Figure 6:**
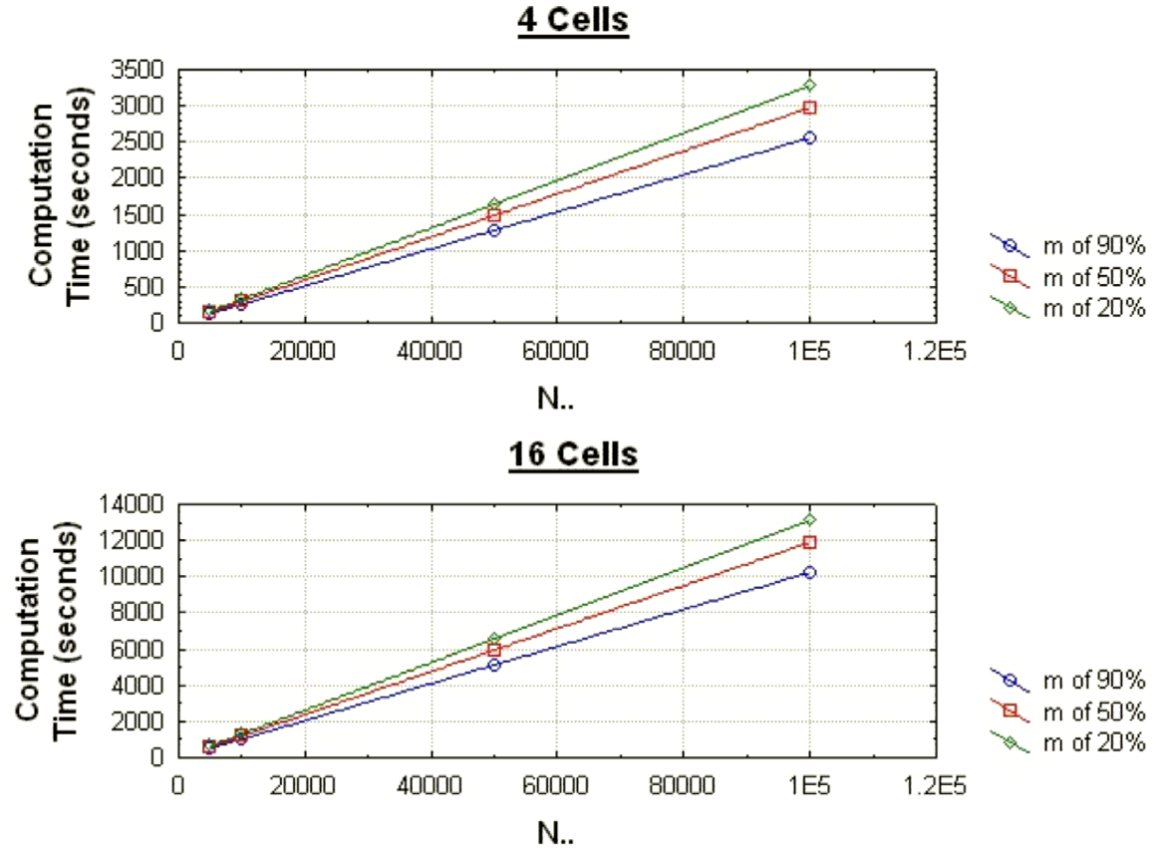
The average computation times for the chi-square test as the proportion of records matching varies for different data set sizes for contingency tables with 4 and 16 cells.

**Figure 7:**
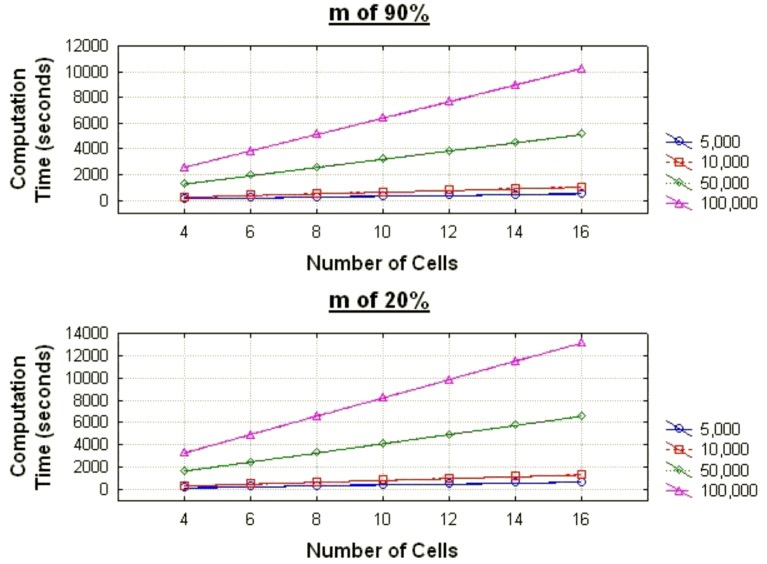
The average computation times for the chi-square test as the number of cells varies for different data set sizes when the proportion of records matching (m) is 90% and 20%.

The graph in Figure 6 shows how the computation time increases linearly as the number of records returned by a query increases. The graph in Figure 7 shows how the computation time increases linearly as the number of cells increases.

The difference between chi-square and odds ratio and relative risk, and their confidence intervals, is negligible because the total computation time is driven by the matching phase rather than the analysis phase. Thus, the computation time for the two latter ones are almost identical as that for chi-square and are not shown here.

## Discussion

### Summary

In this paper we have described a secure protocol for linking records from multiple registries that can be used to monitor the effectiveness of the HPV vaccine. We have shown that this protocol is the only one, thus far, that can meet all five requirements that were derived from actual current constraints for sharing patient information. The protocol can provide specific statistics, and is therefore most useful when the statistics to be computed are known in advance. This fits well with the disease surveillance context where the same statistics would need to be computed on an on-going basis.

The performance of this protocol is acceptable even for large data sets. For example, for a 16 cell contingency table where each of the queries returns 100,000 patients and none of the matches can be performed within an Aggregator, the total computation time is less than 4 hours. This is a worse case assumption, but nevertheless would still be acceptable performance for a surveillance application since the data set will not change at a faster rate than 4 hours. It exhibits a linear increase in computation time as the number of records in the data set grows and as the size of the contingency table grows.

Details on issues that would need to be addressed during the deployment of this protocol in practice, such as dealing with small cells and zero-sized cells, are addressed in Appendix S1.

### HIPAA and the Common Rule

While our initial deployment of this protocol was intended for a Canadian context, its deployment in the US requires special considerations of current legislation and regulations.

The HIPAA Privacy Rule defines two standards for de-identifying health information (45 CFR 164.514(b)). The first one is called the Safe Harbor standard. Under Safe Harbor any unique identifying number, characteristic, or code must be removed from the data set, otherwise it would be considered personal health information.

In our protocol we used a hash function to perform the matching. If a simple hash function was used then it could be reverse engineered using a dictionary attack. For example, if we are using an individual’s social security number and that number is hashed, then an adversary would only need to hash all possible numbers of the same length and compare it to the targeted value until a match is found.

A special type of dictionary attack has also been proposed and utilized to reduce the computation time at the cost of more data storage capacity – the so-called rainbow tables [60]. In this method, a table of hash chains is pre-computed for reversing the hash function by using a reduction function. The chains which create the items in the rainbow table are chains of one way hash functions and reduction functions starting at a certain plaintext, and ending at a certain hashed value. However, only the starting plaintext and ending hashed value are stored in the rainbow table. By comparing against only the stored values a significant reduction in computation can be gained during an attack.

In practice one always adds some randomness to the hash value, as we do in our commutative hash function. This random value is called a “salt” or a “key”. This makes the range of possible values that would need to be checked in a dictionary attack, even with a rainbow table, computationally unattainable in any reasonable amount of time.

Is a hashed value with a salt considered a uniquely identifying code under Safe Harbor?

The HIPAA Privacy Rule requires that “The code or other means of record identification is not derived from or related to information about the individual” *and* that the the covered entity does not use or disclose the code for other purposes or disclose the mechanism for re-identification (45 CFR 164.514(c)). In our protocol we can satisfy the second requirement since the random values used for hashing (the keys) are never shared by the registries. However, a hash value, with or without a salt, is derived from identifiable information and would therefore still be considered personal information under this definition.

The Privacy Rule allows the disclosure of information containing such coded information as a Limited Data Set (45 CFR 164.514(e)). A Limited Data Set would require a data sharing agreement with the data recipient, and the data can only be used for specific purposes: research, public health, or healthcare operations. For users of our protocol, this means that the purpose of the linking and analysis would have to be one of the above. For example, if our protocol is used for public health surveillance or research and a data use agreement is in place between the registries and between the registries and Aggregators, then the requirements for a Limited Data Set would be met.

If our protocol is used for research purposes, then the Common Rule would also apply. Under the Common Rule, which guides IRBs, if the user of the information has no means of getting the key, for example, through an agreement with the other party prohibiting the sharing of keys under any circumstances or through organizational policies prohibiting such an exchange, then this would not be considered human subjects research and would not require an IRB review [61], [62]. In our protocol, if each of the registries has a policy against the sharing of the salt values used, then no IRB approval would be required for the linking project, according to the Common Rule, since the hash values exchanged would not be considered personally identifying information.

This inconsistency between HIPAA and the Common Rule is well documented [63], [64].

However, the Privacy Rule does provide a mechanism for an expert with appropriate statistical knowledge to certify that the data exchanged has a very small risk of re-identification (45 CFR 164.514(a)), at which point it would not be considered personal health information [65]. Therefore, should an expert deem that the (salted) hashed value cannot be reversed and that adequate legal mechanisms exist prohibiting the exchange of the salt represent a very small risk of re-identification, then the data exchanges in our protocol would not be considered identifiable.

In other jurisdictions, such precise prescriptions on the interpretability of coded information are absent, making it easier to argue that the exchange of hashed information (with a salt) for the purpose of matching, with prohibitions on the sharing of that random value, would not constitute an exchange of personal health information.

### Limitations

Our protocol only allows for deterministic matching of patients in the two registries. Therefore, the linking field value that is hashed needs to be a reliable unique identifier across the registries.

We focused on surveillance where the computed statistics are on a contingency table. Where the variable are continuous and cannot be meaningfully discretized into categorical variables, the approach we present will not be suitable.

### Future Work

Extensions of our protocol to allow probabilistic linkage would be expected to result in a higher match rate when there are errors in the variables making up the linking field. For example, the inclusion of names in the set of linking fields would be expected to improve the match rate.

Additional statistics may be added to our protocol to cover more tests that a PHU may wish to use for evaluation of effectiveness or other analytical purposes. We consider two examples of more sophisticated analyses below.

The basic structure of our protocol can also support the computation of population size estimates using a capture-recapture (CR) model. A CR model can estimate the total population of individuals with a particular disease when the registries have an incomplete listing of that population. A secure CR protocol would allow the estimation of population size when the registries are unable to share data. CR models have been used in the biological sciences to estimate the size of animal populations [66], [67], and in epidemiology to estimate birth and death rates [68], [69], as well as the size of diseased populations [70]. The basic principle is that animals are caught on multiple occasions and marked/identified. Using the information on the number of animals caught/not caught on the multiple occasions, a complete capture history of animal capture is known. Methods have been developed to estimate the total population size from such capture histories. When applied to human populations, the overlap across multiple disease registries is used to mimic recaptures. The matching phase of our protocol can compute the number of overlapping individuals between the two registries. An appropriate CR model would then be used to estimate the total population size.

Moving beyond surveillance, multivariate models would need to be constructed to answer questions about whether individuals and populations can be protected against infection and the cervical/anal/oral HPV-associated cancers. They can also be used to better understand HPV vaccine effectiveness through various endpoints [71]–[73], including changes in type-specific HPV prevalence, changes in lesions (via pap test results/colposcopy results), and over a longer period of time, changes in cervical and other HPV-related cancer rates. However, all these may be somewhat impacted by factors such as SES, ethnicity (in the context of accessing care), age of sexual debut, number of sexual partners, parity, smoking, and other factors. It would therefore be important to control for these factors. Future work would need to take our basic surveillance framework and extend it to allow more general secure multivariate modeling, such as for general linear models.

## Supporting Information

Appendix S1 **Description of the commutative hashing function, the protocols for computing other statistics, as well as practical considerations when deploying the protocol.**
(PDF)
