# Supplementary material for: A Protocol for the Secure Linking of Registries for HPV Surveillance
Source: PLoS One. 2012 Jul 2;7(7):e39915. doi: 10.1371/journal.pone.0039915 (PMC3388071; doi:10.1371/journal.pone.0039915)
Supplement: Appendix S1 — Description of the commutative hashing function, the protocols for computing other statistics, as well as practical considerations when deploying the protocol. (PDF) [file pone.0039915.s001.pdf]

Appendix S1

This appendix presents the detailed theory and theoretical results behind our protocol, as well as some of the practical considerations when deploying it in real surveillance settings.

1 A Commutative Hash Function for Private De-duplication

The choice of a commutative hash function is important to ensure that the linking fields, which can identify the patients, cannot be discovered through an attack. In this section we examine the security of a previous matching protocol which uses commutative hash functions, and based on that analysis describe some improvements which we implement in our protocol.

A system was recently developed for the Department of Housing and Urban Development (HUD) for securely tracking and counting domestic violence shelter clients, and was demonstrated in a real setting in the state of Iowa [1]. The problem of securely tracking individuals as they visit multiple sites is similar to our problem of securely matching records in multiple registries.

This HUD system is known as *PrivaMix*. The setup for PrivaMix is similar to ours whereby there are multiple shelters (registeries) sending data to a central planning office (Aggregator) to detect duplicates. The main difference between our protocol and PrivaMix is in what is done with the detected duplicates. In the PrivaMix case the duplicates are used to ensure that domestic violence clients are counted once. In our case duplicates indicate that a patient is in both registries and is counted as a match in a contingency table cell.

PrivaMix uses the Benaloh and de Mare [2] notion of a quasi commutative hash function in the context of their cryptographic accumulator paradigm. Given a public base x and the public product of two large, rigid, primes $n = pq$, a quasi-commutative hash z of a set of values $< y_1 \ldots y_k >$ is computed as follows:

$$z = H(H(\cdots(H(x, y_1), \ldots, y_{k-1}), y_k) = x^{y_1 \cdots y_{k-1} y_k} \bmod n$$

The quasi-commutative property is illustrated by the fact that, given a set of values $< y_1 \ldots y_k >$, the accumulated result z is invariant under arbitrary permutation π :

$$z = H(H(\cdots(H(x, y_{\pi(1)}), \ldots, y_{\pi(k-1)}), y_{\pi(k)}) = x^{y_{\pi(1)} \cdots y_{\pi(k-1)} y_{\pi(k)}} \bmod n$$

At first glance this may seem like a suitable construction for the purposes of secure de-duplication of records (i.e., private set intersection). The primary security goal of an accumulator, however, is to prevent false claims of set membership, and not necessarily to hide the elements themselves. Indeed, the use of the Benaloh/de Mare accumulator in the context of private-set intersection can lead to re-identification via a dictionary attack.

1.1 A Security Analysis of the Shelter Client Tracking Protocol

Below we demonstrate an attack on the PrivaMix de-duplication protocol using the Benaloh/de Mare accumulator. We will use our set-up of matching two registries. Let $R = \{R_1, R_2\}$ be two registries with secret random values r_1 and r_2 respectively. Assume that the linking field is the social security number (SSN) of the patient, and R_1 and R_2 will compute the quasi-commutative hash z of the SSN:

$$z = H(r_2, H(r_1, SSN)) = H(r_1, H(r_2, SSN)) = x^{r_1 r_2 SSN} \bmod n$$

Both registries compute individual hashes for each patient in their respective databases, each sending results to an Aggregator P. De-duplication stems from the fact that a patient whom exists in both registries will generate the same hash as a result of the quasi-commutative property. Since the same hash value will appear in the lists of both registries, P is able to determine that the patient is in both registries.

A dictionary attack is possible and can be mounted independently of the bit length of the random factors r_1 and r_2, allowing an attacker to recover the SSN. Let w represent the total number of valid/possible SSNs. Without loss of generality, we consider an attempt to re-identify the SSNs contained in the partial hash list of registry R_1, with the adversary representing either R_2, P, or any other party able to observe the protocol transcript. Let registry R_1's patient list consist of two social security numbers, SSN_a and SSN_b. Registry R_1 computes the hashes:

$$z_a = H(r_1, SSN_a) = x^{r_1 SSN_a} \bmod n,$$

and

$$z_b = H(r_1, SSN_b) = x^{r_1 SSN_b} \bmod n,$$

and sends them to P, who, in turn, forwards them to R_2.

An adversary, observing z_a and z_b, then builds two dictionaries D_a and D_b as follows:

$$D_a = \{z_a^{SSN_1}, \ldots, z_a^{SSN_w}\},$$

and

$$D_b = \{z_b^{SSN_1}, \ldots, z_b^{SSN_w}\}.$$

The adversary then searches for the $d_i \in D_a$ and the $d_j \in D_b$ such that $d_i = d_j$, which corresponds to:

$$x^{r_1 SSN_a SSN_i} \bmod n = x^{r_1 SSN_b SSN_j} \bmod n$$

The adversary can then conclude that z_a is the hash of social security number SSN_j, and similarly that z_b is the hash of social insurance number SSN_i. More importantly, the bit-length of r_1 has no impact on computational workload of the attack.

Finally, dictionary D_a can be used to efficiently invert additional hashes. For example, to re-identify a third hash $z_c = x^{r_1 SSN_C} \bmod n$, the attacker would simply compute $z_{c'} = (z_c)^{SSN_a} = x^{r_1 SSN_a SSN_c} \bmod n$ and find the $d_{i'} \in D_a$ such that $d_{i'} = z_{c'}$, allowing the attacker to conclude z_c corresponds to the hash of social security number $SSN_{i'}$.

A dictionary of this kind clearly undermines the security claims of this protocol, making privacy dependent on the size of the SSN space, instead of the key/random factor space, as was intended and would be expected.

Is this attack practical? With a social security number space of 9-digits, $w \leq 2^{30}$, building the two dictionaries would require at most 2^{31} modular exponentiations. With an RSA modulus of 1024-bits, a contemporary CPU/GPU might reasonably be expected to compute 2^{10} modular exponentiations per second, suggesting a time scale of 1-2 CPU weeks as an upper bound on building the dictionaries. A standard contemporary multi-core desktop computer, therefore, could compute the dictionaries on the order of a few days, and the task is fully parallelizable. Paying a cloud computing service, for example, could potentially reduce the time to hours or even minutes. Of course, once the dictionaries have been computed, additional re-identifications require only one modular exponentiation and look-up each. The dictionaries require less than 300GB of storage space, which would allow them to fit on a single hard drive.

The existence of an attack against the PrivaMix protocol stems from using a cryptographic primitive—the Benaloh/de Mare accumulator—to fulfill a purpose that it was not designed for. In the following section, we describe alternative constructions that fit our private de-duplication needs, but is not susceptible to the dictionary attack above.

1.2 A Secure Accumulator for Private De-duplication in the RSA Setting

Our first construction relates closely to that of Huberman et al [3], which is considered secure against a passive adversary in the random oracle model. Given a cryptographic function $h()$,e.g., SHA-256, public RSA modulus $n = pq$, secret random factors r_1, r_2 , the hash of message m is computed as follows:

$$z' = H(r_2, H(r_1, m)) = H(r_1, H(r_2, m)) = h(m)^{r_1 r_2} \bmod n$$

The following is a sketch of the security properties:

- Let $c = m^r \bmod n$. It is infeasible to recover m given c , r and n according to the RSA assumption.
- A message raised to a *private* random factor r prevents a chosen plaintext attack. That is, given $c = m^r \bmod n$ and some m' , it is infeasible to determine whether $m' = m$.
- Cryptographic hash $h(m)$ destroys potential linear relationships between messages (with high statistical probability), preventing guessing attacks based on the underlying homomorphic properties. For example, let z_1, z_2 be the commutitive hashes of two SSN's. Suppose an attacker guesses that z_1, z_2 corresponds to SSN's 00000002 and 00000004 respectively. If the cryptographic hash $h()$ is not applied prior to exponentiation, it is easy to see that the attacker can reject this guess if $z_1^2 \neq z_2$. Numerous guesses of this kind can potentially lead to reidentification in a manner similar to the attack presented above. By applying $h()$, the simple linear relationship between the messages is destroyed. We claim it is computationally infeasible to compute $\{a \mid h(m_1)^a \bmod n = h(m_2) \bmod n\}$, and thus infeasible to conduct guessing attacks.

1.3 A Secure Accumulator for Private De-duplication in the Discrete Log Setting

A limitation of the previous construction is the practical difficulty of implementing a secure multi-party protocol to generate an RSA modulus $n = pq$ for which the factors p and q are not known to any protocol participant. Such protocols exist but are inefficient [4, 5].

An alternative construction is to work in the discrete log (DL) setting, which provides two benefits over the previous construction. Firstly, this setting allows the modulus to be selected publically, eliminating the need for a costly setup phase, and secondly, moduli in the DL setting are smaller than RSA moduli at equivalent security levels, allowing for more efficient evaluation.

A construction in this setting offering the desired commutative property is based on the symmetric encryption scheme due Pohlig and Hellman [6]. Let $\mathbb{G}_q$ be a multiplicative subgroup of $\square_p^*$ such that $p = 2q + 1$. Let $e : \square_{q-1} \rightarrow \mathbb{G}_q$ be a bijective encoding function that maps positive integers into $\mathbb{G}_q$. A common choice is $e(m) = (m+1)^2 \bmod p$. Given a cryptographic function $h()$ and message m, the message holder first computes $\hat{m} = e(h(m))$. The quasi-commutative hash of m given secret random factors $\{r_1, r_2\} \in_R \square_{q-1}$, is then defined as [7]:

$$z' = H(r_2, H(r_1, m)) = H(r_1, H(r_2, m)) = \hat{m}^{r_1 r_2} \bmod p \qquad \text{..........................} \ (1)$$

The following is a sketch of the security properties:

- Recovering $\hat{m}$ given z' is equivalent to solving the discrete logarithm problem (DLP),
- A message raised to a *private* random factor prevents a chosen plaintext attack (as described above).
- Cryptographic hash $h()$ prevents guessing attacks (as described above). For two messages $\{m_1, m_2\}$, their encodings $\{\hat{m}_1 = e(h(m_1)), \hat{m}_2 = e(h(m_2))\}$ are uniformly distributed elements in $\mathbb{G}_q$. Consider an a such that $\hat{m}_1^a = \hat{m}_2$. Then $a = \log_{\hat{m}_1}(\hat{m}_2)$. Therefore, any algorithm that can compute $\{a \mid \hat{m}_1^a = \hat{m}_2\}$ can also solve the discrete logarithm problem.

We therefore use the construction in equation (1) in our protocol for matching the identifiers across the registries.

2 Protocols for Other Bivariate Statistics

This section describes the protocols for computing some common bivariate statistics on the linked data. For simplification $\bmod n$ will be ommited from the equations.

We assume that we have the contingency table in Table 1 with the indicated notation.

		**Any HPV**	
		**-ve**	**+ve**
**Ethnicity**	**Aboriginal**	$n_{11} = n_{1,11} + n_{2,11}$	$n_{12} = n_{1,12} + n_{2,12}$
	**White**	$n_{21} = n_{1,21} + n_{2,21}$	$n_{22} = n_{1,22} + n_{2,22}$

Table 1: Notation for computing statistics.

2.1 Chi-Square Test

The chi-square value is given by:

$$\chi^2 = \frac{(n_{11}n_{22} - n_{12}n_{21})^2 (n_{11} + n_{12} + n_{21} + n_{22})}{(n_{11} + n_{12})(n_{21} + n_{22})(n_{12} + n_{22})(n_{11} + n_{21})} \quad \text{.............................. (2)}$$

The two Aggregators will jointly and securely compute the chi-square value, using their private values. Equation (2) can be converted to the following equation:

$$\chi^2 = \frac{\left(\left(n_{1,11}+n_{2,11}\right)\left(n_{1,22}+n_{2,22}\right)-\left(n_{1,12}+n_{2,12}\right)\left(n_{1,21}+n_{2,21}\right)\right)^2\left(\left(n_{1,11}+n_{2,11}\right)+\left(n_{1,12}+n_{2,12}\right)+\left(n_{1,21}+n_{2,21}\right)+\left(n_{1,22}+n_{2,22}\right)\right)}{\left(\left(n_{1,11}+n_{2,11}\right)+\left(n_{1,12}+n_{2,12}\right)\right)\left(\left(n_{1,21}+n_{2,21}\right)+\left(n_{1,22}+n_{2,22}\right)\right)\left(\left(n_{1,12}+n_{2,12}\right)+\left(n_{1,22}+n_{2,22}\right)\right)\left(\left(n_{1,11}+n_{2,11}\right)+\left(n_{1,21}+n_{2,21}\right)\right)}$$

To reach to the final result in equation (2), we will utilize the secure two-party addition and multiplication protocols. The steps are as follows:

1. Aggregator 1 and Aggregator 2 run secure two-party multiplication for the following pairs:

   $n_{1,11}$ and $n_{2,22}$ such that: $n_{1,11} \times n_{2,22} = a_{1,1} + a_{2,1}$

   $n_{1,22}$ and $n_{2,11}$ such that: $n_{1,22} \times n_{2,11} = a_{1,2} + a_{2,2}$

   $n_{1,12}$ and $n_{2,21}$ such that: $n_{1,12} \times n_{2,21} = a_{1,3} + a_{2,3}$

   $n_{1,21}$ and $n_{2,12}$ such that: $n_{1,21} \times n_{2,12} = a_{1,4} + a_{2,4}$

2. Aggregator 1 performs the following computations:

   $a_{1,5} = n_{1,11} \times n_{1,22}$

   $a_{1,6} = n_{1,12} \times n_{1,21}$

   $a_{1,7} = n_{1,11} + n_{1,12} + n_{1,21} + n_{1,22}$

   $a_{1,8} = n_{1,11} + n_{1,12}$

   $a_{1,9} = n_{1,21} + n_{1,22}$

   $a_{1,10} = n_{1,12} + n_{1,22}$

   $a_{1,11} = n_{1,11} + n_{1,21}$

   $a_{1,12} = a_{1,1} + a_{1,2} - a_{1,3} - a_{1,4} + a_{1,5} - a_{1,6}$

3. Aggregator 2 performs the following computations:

   $a_{2,5} = n_{2,11} \times n_{2,22}$

   $a_{2,6} = n_{2,12} \times n_{2,21}$

$a_{2,7} = n_{2,11} + n_{2,12} + n_{2,21} + n_{2,22}$	$a_{2,8} = n_{2,11} + n_{2,12}$
$a_{2,9} = n_{2,21} + n_{2,22}$	$a_{2,10} = n_{2,12} + n_{2,22}$
$a_{2,11} = n_{2,11} + n_{2,21}$	$a_{2,12} = a_{2,1} + a_{2,2} - a_{2,3} - a_{2,4} + a_{2,5} - a_{2,6}$

4. Aggregator 1 and Aggregator 2 run secure two-party addition for the following pairs:

   $a_{1,12}$ and $a_{2,12}$ such that: $a_{1,12} + a_{2,12} = b_{1,1} \times b_{2,1}$

   $a_{1,7}$ and $a_{2,7}$ such that: $a_{1,7} + a_{2,7} = b_{1,2} \times b_{2,2}$

   $a_{1,8}$ and $a_{2,8}$ such that: $a_{1,8} + a_{2,8} = b_{1,3} \times b_{2,3}$

   $a_{1,9}$ and $a_{2,9}$ such that: $a_{1,9} + a_{2,9} = b_{1,4} \times b_{2,4}$

   $a_{1,10}$ and $a_{2,10}$ such that: $a_{1,10} + a_{2,10} = b_{1,5} \times b_{2,5}$

   $a_{1,11}$ and $a_{2,11}$ such that: $a_{1,11} + a_{2,11} = b_{1,6} \times b_{2,6}$

5. Aggregator 1 computes $c_1 = b_{1,1}^2 \times b_{1,2}$ and $d_1 = b_{1,3} \times b_{1,4} \times b_{1,5} \times b_{1,6}$, and sends these to the PHU.
6. Aggregator 2 computes $c_2 = b_{2,1}^2 \times b_{2,2}$ and $d_2 = b_{2,3} \times b_{2,4} \times b_{2,5} \times b_{2,6}$, and sends these to the PHU.

Now $\chi^2 = \frac{c_1 \times c_2}{d_1 \times d_2}$ is computed by the PHU. Since the PHU knows the number of rows and columns, it is relatively straight forward to compute the degrees of freedom and the p-value from that.

2.2 Relative Risk

Relative risk can be computed from the contingency table as follows:

$$r = \frac{n_{11} \times (n_{21} + n_{22})}{n_{21} \times (n_{11} + n_{12})} \qquad \text{.............................. (3)}$$

This can be converted to:

$$r = \frac{(n_{1,11} + n_{2,11})(n_{1,21} + n_{2,21} + n_{1,22} + n_{2,22})}{(n_{1,21} + n_{2,21})(n_{1,11} + n_{2,11} + n_{1,12} + n_{2,12})} = \frac{(n_{1,11} + n_{2,11})(n_{1,21,22} + n_{2,21,22})}{(n_{1,21} + n_{2,21})(n_{1,11,12} + n_{2,11,12})} \qquad \text{.............................. (4)}$$

where $n_{1,21,22} = n_{1,21} + n_{1,22}$, $n_{1,11,12} = n_{1,11} + n_{1,12}$, $n_{2,21,22} = n_{2,21} + n_{2,22}$ and $n_{2,11,12} = n_{2,11} + n_{2,12}$ are locally computed by Aggregator 1 and Aggregator 2. We then follow these steps:

1. By applying secure two-party addition the fraction in equation (14) can be converted to separate and private values for the two Aggregators. Aggregator 1 and Aggregator 2 run secure two-party addition for their following pairs:

   $n_{1,11}$ and $n_{2,11}$ such that: $n_{1,11} + n_{2,11} = a_{1,1} \times a_{2,1}$

   $n_{1,21}$ and $n_{2,21}$ such that: $n_{1,21} + n_{2,21} = a_{1,2} \times a_{2,2}$

   $n_{1,21,22}$ and $n_{2,21,22}$ such that: $n_{1,21,22} + n_{2,21,22} = a_{1,3} \times a_{2,3}$

   $n_{1,11,12}$ and $n_{2,11,12}$ such that: $n_{1,11,12} + n_{2,11,12} = a_{1,4} \times a_{2,4}$

   Thus, the relative risk will be converted to:

$$r = \frac{\left(n_{1,11} + n_{2,11}\right)\left(n_{1,21,22} + n_{2,21,22}\right)}{\left(n_{1,21} + n_{2,21}\right)\left(n_{1,11,12} + n_{2,11,12}\right)} = \frac{\left(a_{1,1} \times a_{1,2}\right)}{\left(a_{1,3} \times a_{1,4}\right)} \times \frac{\left(a_{2,1} \times a_{2,2}\right)}{\left(a_{2,3} \times a_{2,4}\right)}$$

2. Aggregator 1 and Aggregator 2 then compute the two fractions $\frac{b_1}{c_1}$ and $\frac{b_2}{c_2}$, respectively, such that:

$$\frac{b_1}{c_1} = \frac{a_{1,1} \times a_{1,2}}{a_{1,3} \times a_{1,4}} \qquad \frac{b_2}{c_2} = \frac{a_{2,1} \times a_{2,2}}{a_{2,3} \times a_{2,4}}$$

3. Aggregator 1 and Aggregator 2 send their private values, b_1, c_1, b_2, and c_2, to the PHU. Now, PHU computes $r = \frac{b_1 \times b_2}{c_1 \times c_2}$.

2.3 Confidence Interval for Odds Ratio

The confidence interval for odds ratio could be computed as follows:

$$CI_{\ln(OR)} = \ln(\theta) \pm z \times SE \qquad \text{………………… (5)}$$

$$SE = \sqrt{\frac{1}{n_{11}} + \frac{1}{n_{12}} + \frac{1}{n_{11}} + \frac{1}{n_{22}}} \qquad \text{……………. (6)}$$

In equation (5), the confidence interval for the log odds ratio could be computed by the exponentiation of the intervals. The value for z could be derived from the standard normal table.

Equation (6) is converted to the following equation using the Aggregators' private shares. The PHU can perform square root from the final result as follows:

$$SE^2 = \frac{1}{n_{1,11}+n_{2,11}} + \frac{1}{n_{1,12}+n_{2,12}} + \frac{1}{n_{1,21}+n_{2,21}} + \frac{1}{n_{1,22}+n_{2,22}}$$

.... (7)

$$= \frac{A+B+C+D}{\left(n_{1,11}+n_{2,11}\right)\left(n_{1,12}+n_{2,12}\right)\left(n_{1,21}+n_{2,21}\right)\left(n_{1,22}+n_{2,22}\right)}$$

in which:

$$A = \left(n_{1,12}+n_{2,12}\right)\left(n_{1,21}+n_{2,21}\right)\left(n_{1,22}+n_{2,22}\right)$$

$$B = \left(n_{1,11}+n_{2,11}\right)\left(n_{1,21}+n_{2,21}\right)\left(n_{1,22}+n_{2,22}\right)$$

$$C = \left(n_{1,11}+n_{2,11}\right)\left(n_{1,12}+n_{2,12}\right)\left(n_{1,22}+n_{2,22}\right)$$

$$D = \left(n_{1,11}+n_{2,11}\right)\left(n_{1,12}+n_{2,12}\right)\left(n_{1,21}+n_{2,21}\right)$$

To separate the standard error, such that each of the two Aggregators computes a different value, secure two-party addition and multiplication are utilized in the following steps.

1. For equation A, Aggregator 1 and Aggregator 2 run secure two-party multiplication for their following pairs:

   $n_{1,12} \times n_{1,21}$ and $n_{2,22}$ such that: $\left(n_{1,12} \times n_{1,21}\right) \times n_{2,22} = a_{1,1} + a_{2,1}$

   $n_{1,12} \times n_{1,22}$ and $n_{2,21}$ such that: $\left(n_{1,12} \times n_{1,22}\right) \times n_{2,21} = a_{1,2} + a_{2,2}$

   $n_{1,21} \times n_{1,22}$ and $n_{2,12}$ such that: $\left(n_{1,21} \times n_{1,22}\right) \times n_{2,12} = a_{1,3} + a_{2,3}$

   $n_{1,12}$ and $n_{2,21} \times n_{2,22}$ such that: $n_{1,12} \times \left(n_{2,21} \times n_{2,22}\right) = a_{1,4} + a_{2,4}$

   $n_{1,21}$ and $n_{2,12} \times n_{2,22}$ such that: $n_{1,21} \times \left(n_{2,12} \times n_{2,22}\right) = a_{1,5} + a_{2,5}$

   $n_{1,22}$ and $n_{2,12} \times n_{2,21}$ such that: $n_{1,22} \times \left(n_{2,12} \times n_{2,21}\right) = a_{1,6} + a_{2,6}$

2. For equations B, C, and D Aggregator 1 and Aggregator 2 run secure two-party multiplication same as in the previous step to get the output shares, $b_{1,1},\ldots,b_{1,6},c_{1,1},\ldots,c_{1,6},d_{1,1},\ldots,d_{1,6}$, and $b_{2,1},\ldots,b_{2,6},c_{2,1},\ldots,c_{2,6},d_{2,1},\ldots,d_{2,6}$, respectively.

3. For the denominator of equation (7) Aggregator 1 and Aggregator 2 run secure two-party multiplication for their following pairs:

   $n_{1,11} \times n_{1,12} \times n_{1,21}$ and $n_{2,22}$ such that: $\left(n_{1,11} \times n_{1,12} \times n_{1,21}\right) \times n_{2,22} = e_{1,1} + e_{2,1}$

$n_{1,11} \times n_{1,12} \times n_{1,22}$ and $n_{2,21}$ such that: $\left(n_{1,11} \times n_{1,12} \times n_{1,22}\right) \times n_{2,21} = e_{1,2} + e_{2,2}$

$n_{1,11} \times n_{1,21} \times n_{1,22}$ and $n_{2,12}$ such that: $\left(n_{1,11} \times n_{1,21} \times n_{1,22}\right) \times n_{2,12} = e_{1,3} + e_{2,3}$

$n_{1,12} \times n_{1,21} \times n_{1,22}$ and $n_{2,11}$ such that: $\left(n_{1,12} \times n_{1,21} \times n_{1,22}\right) \times n_{2,11} = e_{1,4} + e_{2,4}$

$n_{1,22}$ and $n_{2,11} \times n_{2,12} \times n_{2,21}$ such that: $n_{1,22} \times \left(n_{2,11} \times n_{2,12} \times n_{2,21}\right) = e_{1,5} + e_{2,5}$

$n_{1,21}$ and $n_{2,11} \times n_{2,12} \times n_{2,22}$ such that: $n_{1,21} \times \left(n_{2,11} \times n_{2,12} \times n_{2,22}\right) = e_{1,6} + e_{2,6}$

$n_{1,12}$ and $n_{2,11} \times n_{2,21} \times n_{2,22}$ such that: $n_{1,12} \times \left(n_{2,11} \times n_{2,21} \times n_{2,22}\right) = e_{1,7} + e_{2,7}$

$n_{1,11}$ and $n_{2,12} \times n_{2,21} \times n_{2,22}$ such that: $n_{1,11} \times \left(n_{2,12} \times n_{2,21} \times n_{2,22}\right) = e_{1,8} + e_{2,8}$

$n_{1,11} \times n_{1,12}$ and $n_{2,21} \times n_{2,22}$ such that: $\left(n_{1,11} \times n_{1,12}\right) \times \left(n_{2,21} \times n_{2,22}\right) = e_{1,9} + e_{2,9}$

$n_{1,11} \times n_{1,21}$ and $n_{2,12} \times n_{2,22}$ such that: $\left(n_{1,11} \times n_{1,21}\right) \times \left(n_{2,12} \times n_{2,22}\right) = e_{1,10} + e_{2,10}$

$n_{1,11} \times n_{1,22}$ and $n_{2,12} \times n_{2,21}$ such that: $\left(n_{1,11} \times n_{1,22}\right) \times \left(n_{2,12} \times n_{2,21}\right) = e_{1,11} + e_{2,11}$

$n_{1,12} \times n_{1,21}$ and $n_{2,11} \times n_{2,22}$ such that: $\left(n_{1,12} \times n_{1,21}\right) \times \left(n_{2,11} \times n_{2,22}\right) = e_{1,12} + e_{2,12}$

$n_{1,12} \times n_{1,22}$ and $n_{2,11} \times n_{2,21}$ such that: $\left(n_{1,12} \times n_{1,22}\right) \times \left(n_{2,11} \times n_{2,21}\right) = e_{1,13} + e_{2,13}$

$n_{1,21} \times n_{1,22}$ and $n_{2,11} \times n_{2,12}$ such that: $\left(n_{1,21} \times n_{1,22}\right) \times \left(n_{2,11} \times n_{2,12}\right) = e_{1,14} + e_{2,14}$

4. Aggregator 1 performs the following local computations:

$$g_{1,1} = n_{1,12} \times n_{1,21} \times n_{1,22}$$

$$g_{1,2} = n_{1,11} \times n_{1,21} \times n_{1,22}$$

$$g_{1,3} = n_{1,11} \times n_{1,12} \times n_{1,22}$$

$$g_{1,4} = n_{1,11} \times n_{1,12} \times n_{1,21}$$

$$g_{1,5} = n_{1,11} \times n_{1,12} \times n_{1,21} \times n_{1,22}$$

$$h_{1,1} = \sum_{i=1}^{6} a_{1,i} + \sum_{i=1}^{6} b_{1,i} + \sum_{i=1}^{6} c_{1,i} + \sum_{i=1}^{6} d_{1,i} + \sum_{i=1}^{4} g_{1,i}$$

$$h_{1,2} = g_{1,5} + \sum_{i=1}^{14} e_{1,i}$$

5. Aggregator 2 performs the following local computations:

$$g_{2,1} = n_{2,12} \times n_{2,21} \times n_{2,22}$$

$$g_{2,5} = n_{2,11} \times n_{2,12} \times n_{2,21} \times n_{2,22}$$

$$g_{2,2} = n_{2,11} \times n_{2,21} \times n_{2,22}$$

$$g_{2,3} = n_{2,11} \times n_{2,12} \times n_{2,22}$$

$$g_{2,4} = n_{2,11} \times n_{2,12} \times n_{2,21}$$

$$h_{2,1} = \sum_{i=1}^{6} a_{2,i} + \sum_{i=1}^{6} b_{2,i} + \sum_{i=1}^{6} c_{2,i} + \sum_{i=1}^{6} d_{2,i} + \sum_{i=1}^{4} g_{2,i}$$

$$h_{2,2} = g_{2,5} + \sum_{i=1}^{14} e_{2,i}$$

Therefore, Equation (7) is converted to:

$$SE^2 = \frac{h_{1,1} + h_{2,1}}{h_{1,2} + h_{2,2}}$$

6. Aggregator 1 and Aggregator 2 perform secure two-party addition for the following pairs:

   $h_{1,1}$ and $h_{2,1}$ such that: $h_{1,1} + h_{2,1} = i_1 \times i_2$

   $h_{1,2}$ and $h_{2,2}$ such that: $h_{1,2} + h_{2,2} = j_1 \times j_2$

7. Aggregator 1 and Aggregator 2 send i_1, j_1, i_2, and j_2 to the PHU, respectively. The term $\frac{i_1 \times i_2}{j_1 \times j_2}$ produces the same result as equation (7).

2.4 Confidence Interval for Relative Risk

The confidence interval for relative risk could be computed as follows:

$$CI_{\ln(RR)} = \ln(RR) \pm z \times \sqrt{\frac{n_{12}}{n_{11} \times (n_{11} + n_{12})} + \frac{n_{22}}{n_{21} \times (n_{21} + n_{22})}} \quad \text{................ (8)}$$

Equation (8) is the confidence interval of log relative risk and the confidence interval could be computed by taking the exponentiation of the intervals. The value of z could be derived from the standard normal table. By considering the separate shares of the two aggregators, the value under the square root of equation (8), would be:

$$\frac{n_{1,12} + n_{2,12}}{(n_{1,11} + n_{2,11}) \times (n_{1,11} + n_{2,11} + n_{1,12} + n_{2,12})} + \frac{n_{1,22} + n_{2,22}}{(n_{1,21} + n_{2,21}) \times (n_{1,21} + n_{2,21} + n_{1,22} + n_{2,22})} \quad \text{..... (9)}$$

To compute equation (9), the two Aggregators perform the following steps.

1. Aggregator 1 performs the following local computations:

$$a_{1,1} = n_{1,11} + n_{1,12}$$

$$a_{1,2} = n_{1,21} + n_{1,22}$$

$$a_{1,3} = n_{1,12} \times n_{1,21} \times a_{1,2}$$

$$a_{1,4} = n_{1,22} \times n_{1,11} \times a_{1,1}$$

2. Aggregator 2 performs the following local computations:

$$a_{2,1} = n_{2,11} + n_{2,12} \qquad a_{2,3} = n_{2,12} \times n_{2,21} \times a_{2,2}$$

$$a_{2,2} = n_{2,21} + n_{2,22} \qquad a_{2,4} = n_{2,22} \times n_{2,11} \times a_{2,1}$$

3. Aggregator 1 and Aggregator 2 run secure two-party multiplication for the following pairs:

$(n_{1,12} \times n_{1,21})$ and $a_{2,2}$ such that: $(n_{1,12} \times n_{1,21}) \times a_{2,2} = a_{1,5} + a_{2,5}$

$(n_{1,12} \times a_{1,2})$ and $n_{2,21}$ such that: $(n_{1,12} \times a_{1,2}) \times n_{2,21} = a_{1,6} + a_{2,6}$

$n_{1,12}$ and $(n_{2,21} \times a_{2,2})$ such that: $n_{1,12} \times (n_{2,21} \times a_{2,2}) = a_{1,7} + a_{2,7}$

$(n_{1,21} \times a_{1,2})$ and $n_{2,12}$ such that: $(n_{1,21} \times a_{1,2}) \times n_{2,12} = a_{1,8} + a_{2,8}$

$n_{1,21}$ and $(n_{2,12} \times a_{2,2})$ such that: $n_{1,21} \times (n_{2,12} \times a_{2,2}) = a_{1,9} + a_{2,9}$

$a_{1,2}$ and $(n_{2,12} \times n_{2,21})$ such that: $a_{1,2} \times (n_{2,12} \times n_{2,21}) = a_{1,10} + a_{2,10}$

$(n_{1,22} \times n_{1,11})$ and $a_{2,1}$ such that: $(n_{1,22} \times n_{1,11}) \times a_{2,1} = a_{1,11} + a_{2,11}$

$(n_{1,22} \times a_{1,1})$ and $n_{2,11}$ such that: $(n_{1,22} \times a_{1,1}) \times n_{2,11} = a_{1,12} + a_{2,12}$

$n_{1,22}$ and $(n_{2,11} \times a_{2,1})$ such that: $n_{1,22} \times (n_{2,11} \times a_{2,1}) = a_{1,13} + a_{2,13}$

$(n_{1,11} \times a_{1,1})$ and $n_{2,22}$ such that: $(n_{1,11} \times a_{1,1}) \times n_{2,22} = a_{1,14} + a_{2,14}$

$n_{1,11}$ and $(n_{2,22} \times a_{2,1})$ such that: $n_{1,11} \times (n_{2,22} \times a_{2,1}) = a_{1,15} + a_{2,15}$

$a_{1,1}$ and $(n_{2,22} \times n_{2,11})$ such that: $a_{1,1} \times (n_{2,22} \times n_{2,11}) = a_{1,16} + a_{2,16}$

4. Aggregator 1 performs the following local computations:

$$a_{1,17} = n_{1,11} \times a_{1,1} \times n_{1,21} \times a_{1,2}$$

5. Aggregator 2 performs the following local computations:

$$a_{2,17} = n_{2,11} \times a_{2,1} \times n_{2,21} \times a_{2,2}$$

6. Aggregator 1 and Aggregator 2 run secure two-party multiplication for the following pairs:

$\left(n_{1,11}\times n_{1,21}\times a_{1,1}\right)$ and $a_{2,2}$ such that: $\left(n_{1,11}\times n_{1,21}\times a_{1,1}\right)\times a_{2,2}=a_{1,18}+a_{2,18}$

$\left(n_{1,11}\times n_{1,21}\times a_{1,2}\right)$ and $n_{2,21}$ such that: $\left(n_{1,11}\times n_{1,21}\times a_{1,2}\right)\times n_{2,21}=a_{1,19}+a_{2,19}$

$\left(n_{1,11}\times a_{1,1}\right)$ and $\left(n_{2,21}\times a_{2,2}\right)$ such that: $\left(n_{1,11}\times a_{1,1}\right)\times\left(n_{2,21}\times a_{2,2}\right)=a_{1,20}+a_{2,20}$

$\left(n_{1,11}\times n_{1,21}\times a_{1,2}\right)$ and $a_{2,1}$ such that: $\left(n_{1,11}\times n_{1,21}\times a_{1,2}\right)\times a_{2,1}=a_{1,21}+a_{2,21}$

$\left(n_{1,11}\times n_{1,21}\right)$ and $\left(a_{2,1}\times a_{2,2}\right)$ such that: $\left(n_{1,11}\times n_{1,21}\right)\times\left(a_{2,1}\times a_{2,2}\right)=a_{1,22}+a_{2,22}$

$\left(n_{1,11}\times a_{1,2}\right)$ and $\left(a_{2,1}\times n_{2,21}\right)$ such that: $\left(n_{1,11}\times a_{1,2}\right)\times\left(a_{2,1}\times n_{2,21}\right)=a_{1,23}+a_{2,23}$

$n_{1,11}$ and $\left(n_{2,21}\times a_{2,1}\times a_{2,2}\right)$ such that: $n_{1,11}\times\left(n_{2,21}\times a_{2,1}\times a_{2,2}\right)=a_{1,24}+a_{2,24}$

$\left(n_{1,21}\times a_{1,1}\times a_{1,2}\right)$ and $n_{2,11}$ such that: $\left(n_{1,21}\times a_{1,1}\times a_{1,2}\right)\times n_{2,11}=a_{1,25}+a_{2,25}$

$\left(a_{1,1}\times n_{1,21}\right)$ and $\left(a_{2,2}\times n_{2,11}\right)$ such that: $\left(a_{1,1}\times n_{1,21}\right)\times\left(a_{2,2}\times n_{2,11}\right)=a_{1,26}+a_{2,26}$

$\left(a_{1,1}\times a_{1,2}\right)$ and $\left(n_{2,11}\times n_{2,21}\right)$ such that: $\left(a_{1,1}\times a_{1,2}\right)\times\left(n_{2,11}\times n_{2,21}\right)=a_{1,27}+a_{2,27}$

$a_{1,1}$ and $\left(n_{2,11}\times n_{2,21}\times a_{2,2}\right)$ such that: $a_{1,1}\times\left(n_{2,11}\times n_{2,21}\times a_{2,2}\right)=a_{1,28}+a_{2,28}$

$\left(n_{1,21}\times a_{1,2}\right)$ and $\left(n_{2,11}\times a_{2,1}\right)$ such that: $\left(n_{1,21}\times a_{1,2}\right)\times\left(n_{2,11}\times a_{2,1}\right)=a_{1,29}+a_{2,29}$

$n_{1,21}$ and $\left(n_{2,11}\times a_{2,1}\times a_{2,2}\right)$ such that: $n_{1,21}\times\left(n_{2,11}\times a_{2,1}\times a_{2,2}\right)=a_{1,30}+a_{2,30}$

$a_{1,2}$ and $\left(n_{2,11}\times n_{2,21}\times a_{2,1}\right)$ such that: $a_{1,2}\times\left(n_{2,11}\times n_{2,21}\times a_{2,1}\right)=a_{1,31}+a_{2,31}$

7. Aggregator 1 performs the following local computations:

$$b_1=\sum_{i=3}^{16}a_{1,i} \qquad c_1=\sum_{i=17}^{31}a_{1,i}$$

8. Aggregator 2 performs the following local computations:

$$b_2=\sum_{i=3}^{16}a_{2,i} \qquad c_2=\sum_{i=17}^{31}a_{2,i}$$

Equation (9) can be represented as:

$$\frac{b_1 + b_2}{c_1 + c_2} \quad \text{.................} \ (10)$$

9. Aggregator 1 and Aggregator 2 perform secure two-party addition for the following pairs:

   b_1 and b_2 such that: $b_1 + b_2 = d_1 \times d_2$

   c_1 and c_2 such that: $c_1 + c_2 = g_1 \times g_2$

10. Aggregator 1 and Aggregator 2 send d_1, g_1, d_2, and g_2 to the PHU, respectively. The term $\frac{d_1 \times d_2}{g_1 \times g_2}$ produces equation (10).

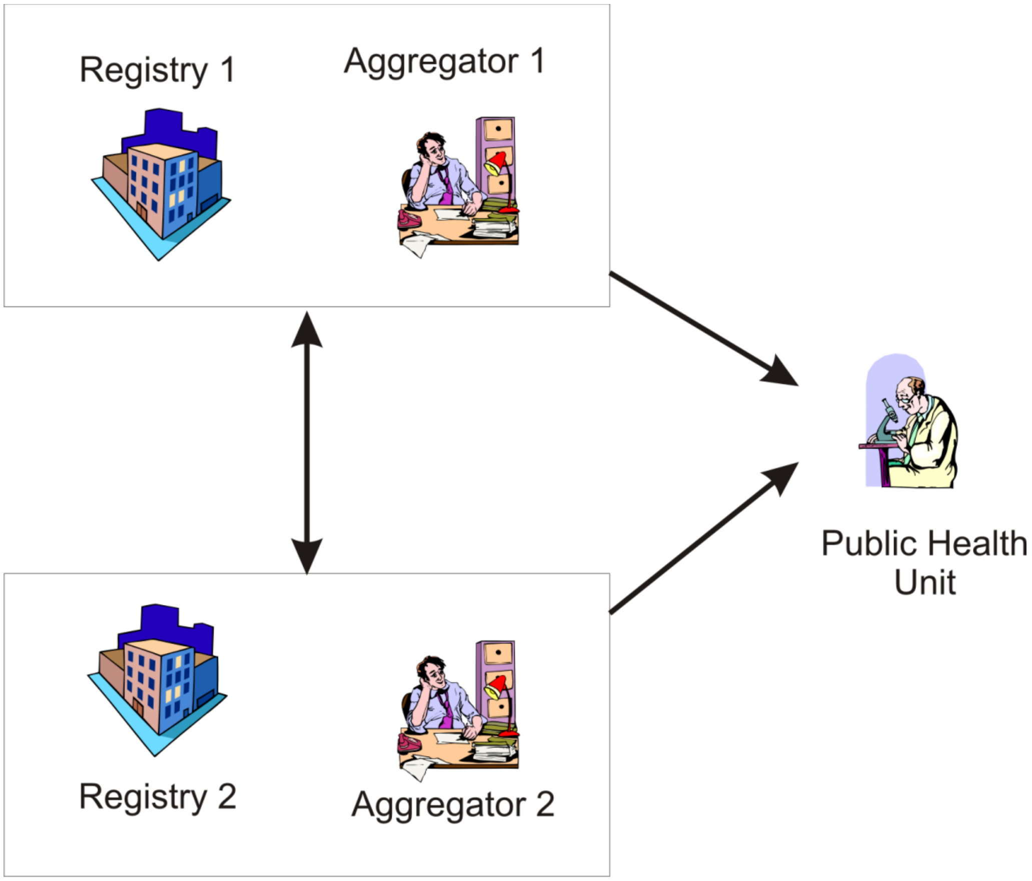

Figure 1: Each Aggregator can be incorporated within the registry to make a single node.

3 Security Analysis

This protocol assumes that the Aggregators are semi-trusted. This means that they would not have access to any raw linking values nor sensitive values about the patients. Nor would they be able to determine identity from small cells because any individual Aggregator would not have the complete cell count for any cell in the contingency table. This also means that if an Aggregator is compromised by an adversary, the adversary would not be able to get access to personal health information. The only way an Aggregator would know the correct cell count in plaintext is if there is collusion between the two Aggregators.

The registries do not share any secret keys and a compromise at one registry would not affect the other one. This means that the registries do not need to trust each other. Furthermore, it is not possible for a registry to gain additional information about any of its patients through this protocol. For example, it is not possible for a registry to learn if any of its patients is in the other registry.

Collusion between a registry and an Aggregator would not compromise the security of the protocol since this would still not allow either of them to know the complete cell count. Furthermore, collusion between an Aggregator and the PHU would not create a risk because they would not be able to reverse engineer the cell counts.

The PHU does not get raw data nor the actual contingency table. By obtaining only the statistics the PHU can achieve its surveillance objectives without having access to patient identities.

We use two basic protocols, secure two-party addition and multiplication. The security of these protocols has already been established [8].

Under certain circumstances it may be possible to reduce the number of nodes. If both registries are population registries (i.e., everyone in the population is included in these registries), then it would be possible to combine each registry with an aggregator into a single node as illustrated in Figure 1. In practice, because collusion between a registry and the Aggregator does not compromise the security of the protocol it would be safe to combine them into a single node. This would also reduce the number of nodes required to deploy the protocol by 50%. With such a deployment, no additional physical nodes are required to operationalize the protocol and no independent third parties are necessary (effectively, each registry acts as an sTTP for the other registry).

With a combined node and population registries, a registry will not learn whether any of its patients is in the other registry because all patients are. If either registries has a subset of the population, then the registry and Aggregator cannot be combined into a single node because one of the registries would learn something new, at least for some of the patients. This new information would be that a patient exists in the other registry.

4 Theoretical Performance Evaluation

The challenge with secure computation protocols is that they are slower than non-secure ones. This makes the assessment of performance an important determinant of their practicability.

The performance of this protocol is driven by computation time and communication time. The communication time will be a function of the network latency among the parties; therefore we will only consider the number of messages exchanged. We assume that each value sent is a message. This is a conservative assumption as often multiple values would be bundled together in a single message.

4.1 Matching Phase

We will use the notation N_{1+} to denote the number of patients who match a query Q_{1+}.

4.1.1 Communication Costs

The computation of the commutative hash for any single patient in a registry requires three messages. Therefore, the theoretical total number of messages that will be exchanged for the four queries required

for a 2x2 contingency table are: $3\left(N_{1+}+N_{2+}+N_{+1}+N_{+2}\right)$. Although in practice, each registry will respond with all of the hashed values per query in one message, therefore 12 messages would be transmitted.

4.1.2 Computation Time

Let τ be the time it takes to perform a single commutative hash. The total computation time to get all of the hash values for the patients would be: $2\tau\left(N_{1+}+N_{2+}+N_{+1}+N_{+2}\right)$. The value of τ needs to be estimated empirically.

The actual matching consists of two steps: (a) matching the hashed values within each Aggregator, and (b) those that do not match are reconciled among the two Aggregators.

Each registry randomly sends half its hashed values to each Aggregator. Therefore Aggregator 1 will have on average $\frac{N_{1+}}{2}$, $\frac{N_{+2}}{2}$, $\frac{N_{+1}}{2}$, and $\frac{N_{2+}}{2}$ patient hashes, and Aggregator 2 will have a similar number of patient hashes for each of the cells.

Matching the hashed values within the Aggregator (step a above) can be done quite efficiently. If the Aggregator is matching $\frac{N_{1+}}{2}$ values with $\frac{N_{+2}}{2}$ values, then only $\min\left(\frac{N_{1+}}{2},\frac{N_{+2}}{2}\right)$ comparisons are required if the two lists are ordered. The complexity of a merge sort is in the order of $\frac{N_{1+}}{2}\times\log\left(\frac{N_{1+}}{2}\right)$ and $\frac{N_{+2}}{2}\times\log\left(\frac{N_{+2}}{2}\right)$ for each of the two hash value lists [9].

Let ${}_1m_{1+,+2}$ denote the number of hashed values that can be matched by Aggregator 1 when it matches the patient hash values that are returned by Q_{1+} and Q_{+2} in step (a). This means that $\frac{N_{1+}}{2}-{}_1m_{1+,+2}$ and $\frac{N_{+2}}{2}-{}_1m_{1+,+2}$ hashed values will need to be reconciled with Aggregator 2. With ordered lists this can be performed in $\min\left(\frac{N_{1+}}{2}-{}_1m_{1+,+2},\frac{N_{+2}}{2}-{}_2m_{1+,+2}\right)$ and $\min\left(\frac{N_{+2}}{2}-{}_1m_{1+,+2},\frac{N_{1+}}{2}-{}_2m_{1+,+2}\right)$ comparisons.

If the value of ${}_1m_{1+,+2}=0$ and ${}_2m_{1+,+2}=0$ then this is the maximum number of hash values that need to be reconciled in step (b). On the other hand if ${}_1m_{1+,+2}=\frac{N_{1+}}{2}$ and ${}_2m_{1+,+2}=\frac{N_{+2}}{2}$ then no reconciliation between the two aggregators is needed.

Because reconciliation requires a secure two party addition, this will be more computationally expensive than step (a). Let α be the time it takes to perform an encryption or decryption. It has been shown that secure two-party addition and multiplication each requires 3α time [8]. However, because the encryptions are done separately and in parallel by the two parties, the elapsed computation time would be 2α. Therefore, the total computation time for the first cell would be:

$$2\alpha\times\left[\min\left(\frac{N_{1+}}{2}-{}_1m_{1+,+2},\frac{N_{+2}}{2}-{}_2m_{1+,+2}\right)+\min\left(\frac{N_{+2}}{2}-{}_1m_{1+,+2},\frac{N_{1+}}{2}-{}_2m_{1+,+2}\right)\right].$$

The general scheme described above would apply to all of the remaining cells. For each secure 2-party addition and multiplication two messages need to be transmitted. In practice, when matching each Aggregator will send all of the values that need to be matched at once rather than one by one. Therefore the total number of messages transmitted will be 2.

4.2 Analysis Phase

The time to perform the computation of statistics will be dominated by the encryption/decryption time. Therefore, we focus on those as the main performance measurements.

The computation of the chi-square value requires a fixed $10 \times 2\alpha$ computation time for each cell. The computation of odds ratio requires a fixed $4 \times 2\alpha$ computation time per cell. The relative risk value requires a fixed $4 \times 2\alpha$ computation time as well. As can be seen, the actual computation time is not related to the size of the data set.

The total number of messages that would need to be transmitted is 20 for the chi-square test, and 8 for the odds ratio and relative risk calculations per cell.

4.3 Deployment

4.3.1 Deployment Requirements

When deploying our protocol, a number of issues need to be addressed:

- **Interactive Use.** The protocol we have presented assumes that the PHU would interactively send queries to the registries. Therefore, the registries need to be available continuously to respond to such queries.
- **Unique identifiers.** Since the protocol assumes deterministic matching, a reliable set of unique identifiers or linking variables are required.
- **Certificate authority.** The basic protocol does not have a requirement for a centralized certificate issuing authority. However, in practice such an authority would be needed to implement digital signatures and secure communication among the parties. Current commercially available certificate authorities can be used for that purpose.
- **Authentication and digital signatures.** Each registry would need to set-up appropriate credentials for the other registries that it wishes to collaborate with. In addition, digital signatures would be set-up so that the messages among the parties can be signed. A discussion of digital signatures in the context of secure multi-party computation protocols for public health is provided elsewhere [10].

While we described only three statistics on a contingency table, by following the same principles that we describe here, one can extend this to other types of statistics on contingency tables.

4.3.2 Audit Mechanism

With a 2x2 contingency table there are four unknown values. If the PHU obtains four statistics on the same contingency table then it would be possible to analytically reverse engineer the values of each of the cells in the table. To mitigate against such a risk an automated audit mechanism needs to be put in place. This would allow a maximum of $C-1$ statistics on the same contingency table, where C is the number of cells in the table. A contingency table is characterized by the response categories in each of its dimensions and the date range. This assumes that the data at the registries is static. If the data at the registries is dynamic then the limit would be $C-1$ for each data snapshot. Such an audit mechanism would not protect against collusion among users. For example, one user may request the maximum of three statistics and another user may request the remaining statistic, and then together they can compute the cell values.

An audit mechanism does not address two other scenarios: (a) collusion among PHUs who share statistics on the same table where each PHU is below the number of statistics threshold, and (b) PHUs that publish statistics. Risks from collusion can be addressed through data sharing agreements with the PHUs. Active collusion would be a deliberate violation of a contract and cannot happen inadvertently. Publishing statistics would also be prohibited by such an agreement.

4.3.3 Dishonest Parties

Our model assumed that the parties are semi-trusted. With the deployment scenario we described above this requires a registry to be semi-trusted by the other registry. In situations where such trust may not be warranted or the registry wishes to establish stronger guarantees, one can employ zero-knowledge proofs to extend the current protocol, and this would protect against malicious parties [11].

4.3.4 PHU and Registry Complexity

The proposed protocol will only be implemented in practice if it is simple to use by the PHU. Most of the complexity in the protocol would be embedded within the software that implements the registry and Aggregator functions. For registries they would only need to set-up the hardware and software, connect that to the source database(s), indicate which fields they are willing to share, and configure the accounts for the other registries that they are willing to share data with. Once the software set-up is complete, the secure linking and computation of statistics is transparent to the registry and does not require further interventions.

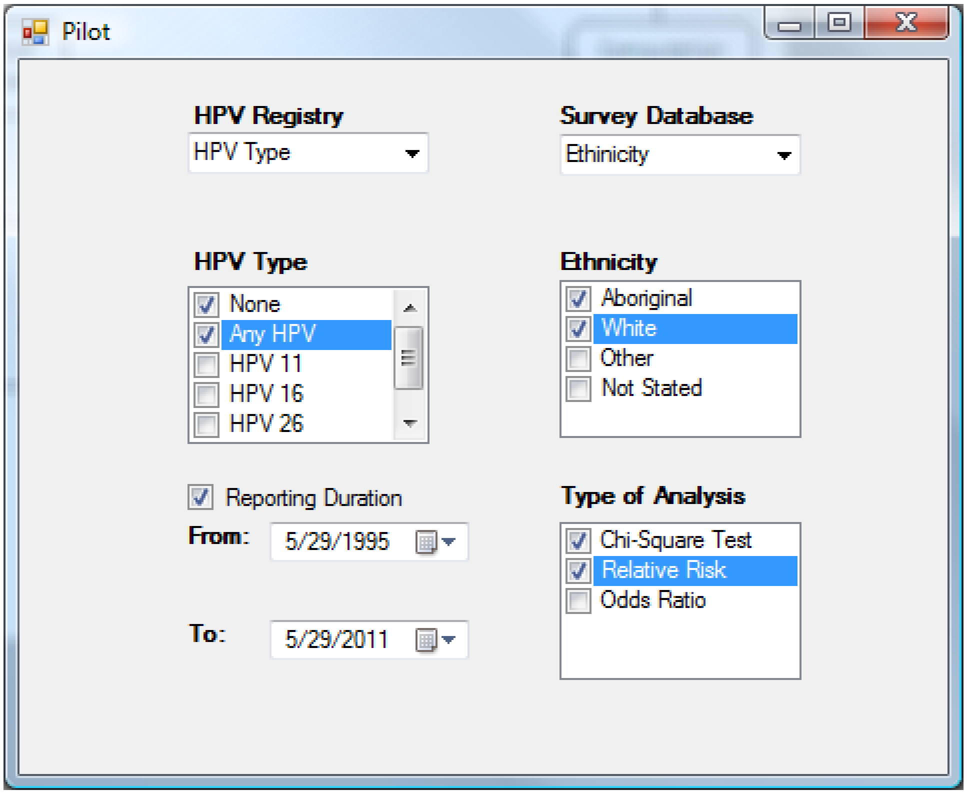

Figure 2: A screen shot showing the interface that the PHU would see based on our pilot implementation.

As an illustration, the end-user would interact with the system through an interface shown in Figure 2. Here the PHU selects the variables in each registry they have access to and the response categories to

be included in the contingency table. The date interval and the particular statistics requested would also be selected.

4.4 Zero Cells in Contingency Table

If any of the cells have a zero value, then the result of secure two-party addition will be zero. This would reveal the existence of a zero cell in the contingency table during the computation of statistics to one of the Aggregators and subsequently to the PHU. It is not uncommon for secure computation protocols to reveal a zero input to one of the two parties [12]. However, in our case the exposure of a zero cell would compromise our requirements.

To address this problem it is necessary to add a small constant value to each cell sum, such as 5×10^{-11}. The original equations in the main paper for each of the aggregators were:

$$\begin{aligned} n_{1,12} &= |S_1| + c_1 \\ n_{2,12} &= |S_2| + c_2 \end{aligned} \qquad \text{............................ (11)}$$

would be modified to:

$$\begin{aligned} n_{1,12} &= |S_1| + c_1 + 5\times10^{-11} \\ n_{2,12} &= |S_2| + c_2 + 5\times10^{-11} \end{aligned} \qquad \text{............................ (12)}$$

Because all of the computations thus far have been on integers, we would need to change the scale for the two-party addition and multiplication sub-protocols to work on real numbers instead to accommodate the addition of a small fraction to the cell counts. The modified building block protocols for real numbers are described below.

4.4.1 Protocols with Scaling

Scaling has to be utilized in these cases. Following are the ways we follow in the two main building blocks, secure addition and multiplication, and the technique is the same in other building blocks. Here without loss of generality we work on two-party case and general case would be the same.

4.4.1.1 Secure Two-Party Addition

Suppose there are two inputs a_1 and a_2 (real numbers) owned by the two parties P_1 and P_2, respectively. The parties agree on an integer number, $s = s_1 + s_2$, as a scale to convert their numbers into integers. Therefore, each party would do the scaling as follows:

$$b_1 = a_1 \times 10^s \qquad\qquad b_2 = a_2 \times 10^s$$

Then they run the secure addition protocol to obtain two private outputs c_1 and c_2, such that:

$$b_1 + b_2 = c_1 \times c_2$$

Now we have:

$$c_1 \times c_2 = (a_1 + a_2) \times 10^s$$

Therefore, the final outputs, d_1 and d_2, would be:

$$d_1 = \frac{c_1}{10^{s_1}} \qquad d_2 = \frac{c_2}{10^{s_2}}$$

Using scaling the final equation is:

$$a_1 + a_2 = d_1 \times d_2$$

4.4.1.2 Secure Two-Party Multiplication

Suppose there are two inputs a_1 and a_2 (real numbers) owned by the two parties P_1 and P_2, respectively. The parties agree on an integer number, s, as a scale to convert their numbers to integer. Therefore, each party do the scaling as follows:

$$b_1 = a_1 \times 10^s \qquad b_2 = a_2 \times 10^s$$

Then they run the secure multiplication protocol to obtain two private outputs c_1 and c_2, such that:

$$b_1 \times b_2 = c_1 + c_2$$

Now we have:

$$c_1 + c_2 = \left(a_1 \times 10^s\right) \times \left(a_2 \times 10^s\right)$$

Therefore, the final outputs, d_1 and d_2, would be:

$$d_1 = \frac{c_1}{10^{2\times s}} \qquad d_2 = \frac{c_2}{10^{2\times s}}$$

Using scaling the final equation is:

$$a_1 \times a_2 = d_1 + d_2$$

4.4.2 Evaluation of Modified Building Blocks

In practice, the addition of a small constant to the cell counts will result in a negligible effect on the computation of statistics. To illustrate the size of this error we computed the effect on the chi-square test for sizes of tables up to 60 cells by 60 cells using randomly generated data ranging from 68,000 to 98,000 individuals in the table. The mean error and mean square error are shown in Table 2. In Table 3 we show a set of cases where cell counts are limited to only 5. Here the constant error represents a larger proportion of the count than for cells with large numbers. As can be seen, the error is quite small and would have negligible impact on the analysis results. We obtained similar results for the other statistics.

Tables size	Average difference	MSE
4x4	10^{-10}	10^{-21}
10x10	10^{-8}	10^{-19}
20x20	10^{-7}	10^{-17}
30x30	10^{-6}	10^{-16}
60x60	10^{-5}	10^{-14}

Table 2: Accuracy of chi-square statistic with randomly generated data and a small constant added to cell counts for 100 iterations. MSE is the mean square error.

Tables size	Average difference	MSE
4x4	10^{-9}	10^{-20}
10x10	10^{-8}	10^{-19}
20x20	10^{-7}	10^{-17}
30x30	10^{-6}	10^{-17}
60x60	10^{-6}	10^{-15}

Table 3: Accuracy of chi-square statistic with randomly generated data and a small constant added to small cell counts (a maximum of 5 in each cell) for 100 iterations. MSE is the mean square error.

4.5 Small Cells and Statistical Testing

One general issue regarding the stability of the final results for statistics using the contingency table is when the values of one or more cells are very small, say 5 or less. In this situation the chi-square will have reduced accuracy because of the small samples being used for estimation. The Aggregator, using their private input shares for the cells, can play a critical function in detecting this and alert the PHU. To do this, an Aggregator can run secure comparison on their private shares for each cell. Secure comparison would compare the count with a specific threshold and detect the occurrence of this condition without knowing each other's private values for the cells. Suppose, the Aggregators want to compare n_{11} with the constant threshold number K, and each of them has her own private share as follows:

$$n_{11} = n_{1,11} + n_{2,11}$$

They can run the following steps for the secure comparison:

1. Aggregator 1 encrypts her private value, $n_{1,11}$, and sends $E(n_{1,11})$ to Aggregator 2.
2. Aggregator 2 generates a private random number, r_2, encrypts that value and the constant value, K, and sends $\left(E(n_{1,11}) \times E(n_{2,11}) \times E(-K)\right)^{r_2}$ to Aggregator 1.
3. Aggregator 1 decrypts the received value and compares it with zero. If the decrypted value is less than zero, it means that $n_{11} < K$.

This sub-protocol will be performed by the Aggregators for their private values of each cell. If small cells are detected then they can inform the PHU as part of providing the results.

4.6 Differential Privacy: An Alternative Privacy-Preserving Mechanism?

A possible alternative approach that we considered for the disclosure of statistics for the purpose of HPV surveillance is differential privacy [13]. Below we first briefly describe differential privacy, how it can be used within our protocol, and then explain why it would not be appropriate in this context.

Differential privacy requires that the answer to any query be "probabilistically indistinguishable" with or without a particular row in the dataset. In other words, For any two datasets D and D', drawn from a population $\wp$, that differ in exactly one record, if K_f is a randomized function used to respond to an arbitrary query f, then K_f gives ε-differential privacy if $\Pr[K_f(D)=s] \leq \mathrm{e}^{\varepsilon} \Pr[K_f(D')=s]$. Some authors interpret this as: "knowledge gain ratio from one data set over the other" [14]. Differential privacy requires that the knowledge gain is less than e^{ε}. Even if an individual removed her data from the data set, no output would become significantly more or less likely [13]. The parameter ε is public. Although there is no principled basis for choosing ε, values are typically set at 0.01, or 0.1, and sometimes $\ln 2$ or $\ln 3$.

In order to satisfy differential privacy in a query response situation, it has been common to use Laplace distributed noise [15]: if r is the correct answer to a query f on a dataset $D : f(D) = r$, then a differentially private mechanism would output the response $r + y$, where y is the noise drawn at random from a Laplace distribution with mean 0 and scale $\Delta f / \varepsilon$. Δf represents the maximum value for $\| f(D) - f(D') \|$ for all $D, D' \in \wp$ differing in one row. In other words, it is the maximum difference in the values of $f(D)$ when exactly one row of data is changed. Δf is the global sensitivity of the query f over domain $\wp$, it is independent of the dataset and one must consider all datasets $D \in \wp$ to determine its value.

If we treat a contingency table as a histogram, its sensitivity is 1 (since the addition or removal of any database row affects the counts in one location by at most 1). Hence we can add independently generated noise to the 2^k cells of the contingency table with distribution $Laplace\left(0, 1/\varepsilon\right)$, where k is the number of variables (dimensions of the contingency table) and if we assume all variables are binary (non-binary variables can be represented as multiple binary variables).

A Laplace random variable can be represented as the difference of two independent and identically distributed exponential random variables. So if X_1 and X_2 are two independent variables that are exponentially distributed with parameter *a*, then $X_1 - X_2$ is a Laplace distribution with parameters $\left(0, 1/a\right)$. In our case $1/a$ is $1/\varepsilon$, and therefore one Aggregator can add noise with distribution *exponential(a)* and the other Aggregator can add noise with distribution *–exponential(a)* in the equations computing the cell sizes. The original equations for each of the aggregators:

$$n_{1,12} = |S_1| + c_1$$
$$n_{2,12} = |S_2| + c_2 \qquad \ldots\ldots\ldots (13)$$

would be modified to:

$$n_{1,12} = |S_1| + c_1 + exponential(0, \varepsilon)$$
$$n_{2,12} = |S_2| + c_2 - exponential(0, \varepsilon)$$
.... (14)

This way each cell in the contingency table is perturbed with appropriate noise. The full contingency table can then be dislcosed directly to the PHU. The PHU can perform as many statistics as they wish on the table and these would all be differentially private statistics.

The problem with this approach is that the amount of noise generated when computing marginals could be large [13]. For example, the variance of the 1-way table described by any one variable is $2^{k-1}\varepsilon^{-2}$ [13].

Alternatively, if low order marginals are sufficient for the PHU, then an sTTP can release a set of say M marginals. Each marginal has sensitivity 1, hence the amount of noise added to each of the released marginals should be proportional to M / ε, when n is large compared to M / ε, the authors suggest that this will yield good accuracy. However, with this approach, there will be inconsistencies between the different marginals as noise is added independently.

Another (non-efficient) alternative introduced by [16] is to construct a synthetic database that is positive and integral and that preserves all low order marginals up to a small error using Fourier transforms. However, an evaluation of this mechanism using three real-life data sets concluded that it is not suitable, especially for large and sparse tables [17].

Therefore, differential privacy at this point is not seen as providing an alternative practical basis for an HPV surveillance protocol. Additional details on the limitations of differential privacy for applications in healthcare are summarized elsewhere [18].

5 References

1. Sweeny L. Demonstration of a Privacy-preserving System that Performs an Unduplicated Accounting of Services Across Homeless Programs. 2008; Carnegie Mellon University, Data Privacy Lab Working Paper 902 (US Government Release).
2. Benaloh J, de Marc M. One-way accumulators: A decentralized alternative to digital signatures. Advances in Cryptography (EUROCRYPT). 1994.
3. Huberman B, Franklin M, Hogg T. Enhancing privacy and trust in electronic communities. Proceedings of the 1st ACM Conference on Electronic Commerce. 1999.
4. Boneh D, Franklin M. Efficient generation of shared RSA keys. Journal of the ACM (JACM), 2001; 48(4):702-722.
5. Gilboa N. Two Party RSA Key Generation. Crypto '99, Lecture Notes in Computer Science vol 1666. 1999.
6. Pohlig S, Hellman M. An Improved Algorithm for Computing Logrithms over GF(p) and its Cryptographic Significance. IEEE Transactions on Information Theory, 1978; 24(1):106-110.
7. Agrawal R, Evmievski A, Srikant R. Information sharing across private databases. Proceedings of the 2003 ACM SIGMOD International Conference on Management of Data. 2003.
8. Samet S, Miri A. Privacy-Preserving Bayesian Network for Horizontally Partitioned Data. The 2009 IEEE International Conference on Information Privacy, Security, Risk and Trust (PASSAT2009). 2009: Vancouver, Canada. p. 9-16.
9. Goldreich O. Computational Complexity. 2008: Cambridge University Press.
10. El Emam K, Hu J, Mercer J, Peyton L, Kantarcioglu M, Malin B, Buckeridge D, Samet S, Earle C. A Secure Protocol for Protecting the Identity of Providers When Disclosing Data for Disease Surveillance. Journal of the American Medical Informatics Association, 2011; 18(3):212-217.
11. Kantarcioglu M, Kardes O. Privacy preserving data mining in the malicious model. International Journal of Information and Computer Security, 2009; 2(4):353-375.

12. Clifton C, Kantarcioglou M, Vaidya J, Lin X, Zhu N. Tools for privacy preserving distributed data mining. ACM SIGKDD Explorations, 2002; 4(2):28-34.
13. Dwork C. Differential privacy: A survey of results. Proceedings of the 5th International Conference on Theory and Applications of Models of Computation. 2008.
14. Abowd J.M., Vilhuber L. How protective are synthetic data. Privacy in Statistical Databases. 2008.
15. Dwork C, McSherry F, Nissim K, Smith A. Calibrating noise to sensitivity in Private data analysis. Third Theory of Cryptography Conference. 2006.
16. Barak B, Chaudhuri K, Dwork C, Kale S, McSherry F, Talwar K. Privacy, Accuracy, and Consistency Too: A Holistic Solution to Contingency Table Release. Proceedings of the twenty-sixth ACM SIGMOD-SIGACT-SIGART symposium on Principles of database systems. 2007.
17. Fienberg S, Rinaldo A, Yang X. Differential Privacy and the Risk Utility Tradeoff for Multi-dimensional Contingency Tables. Privacy in Statistical Databases. 2010.
18. Dankar F, El Emam K. The Application of Differential Privacy to Health Data. The 5th International Workshop on Privacy and Anonymity in the Information Society (PAIS). 2012.
